# Mastitis and Mammary Abscess Management Audit (MAMMA) in the UK and Ireland

**DOI:** 10.1093/bjs/znad333

**Published:** 2023-10-31

**Authors:** Alona Courtney, Jonathon Clymo, Ruth Parks, Alexander Wilkins, Ruth Brown, Rachel O’Connell, Rajiv Dave, Marianne Dillon, Hiba Fatayer, Rachel Gallimore, Ashu Gandhi, Matthew Gardiner, Victoria Harmer, Lyndsey Hookway, Gareth Irwin, Charlotte Ives, Helen Mathers, Juliette Murray, D Peter O’Leary, Neill Patani, Sophie Paterson, Shelley Potter, Ruth Prichard, Giovanni Satta, T G Teoh, Paul Ziprin, Stuart McIntosh, Michael R Boland, Daniel Richard Leff, the MAMMA Research Collaborative, Ahmed Ahmed, Ahmed Shalaby, Akanksha Kiran, Alexander Boucher, Alexander Ribbits, Alexandra Tenovici, Alice Chambers, Alice Lee, Alison Bate, Amanda Koh, Anita Sharma, Anjelli Wignakumar, Anna Fullard, Anna Isaac, Anneliese Lawn, Aonghus Ansari, Arjuna Brodie, Arthika Surendran, Ashvina Segaran, Ayesha Abbasi, Azel Regan, Badr Al-Khazaali, Bahar Mirshekar-Syahkal, Bahaty Riogi, Benjamin Patel, Brenda Muntean, Buket Ertansel, Candice Downey, Carolyn Cullinane, Catherine Rossborough, Charlotte Kallaway, Chiara Sirianni, Chwanrow Baban, Ciaran Hollywood, Clare Roger, Colin McIlmunn, Deeksha Arora, Despoina Chatzopoulou, Diya Mirghani, Ed Babu, Eilidh Bruce, Eiman Khalifa, Elaf Osman, Eleftheria Kleidi, Eleni Ntakomyti, Emma Kellett, Erum Najeeb, Evangelos Mallidis, Fiona Rutherford, Francesca Malcolm, Francesk Mulita, Gabriella Marchitelli, Gemma Hughes, George Neelankavil Davis, Georgios Karagiannidis, Ghadah Alyahya, Ghassan Elamin, Giovanni Santoro, Goran Ahmed, Grace Knudsen, Grant Harris, Gwen Bromley, Hana Esack, Hannah Markey, Harry Yeuk Hei Lei, Heather Pringle, Hedwige Nathaniel, Henry D Robb, Hytham K S Hamid, Ibrahim Elzayat, Ishita Handa, Jaideep Rait, Javeria Iqbal, Jayan George, Jenna Morgan, Jennifer Long, Jenny Banks, Jih Dar Yau, Joanna Stringer, Joey Fong, Joseph Maalo, Josh Marston, Joshua Silva, Julia Massey, Katharine Kirkpatrick, Katherine De Rome, Katherine Fairhurst, Katie Campbell, Katie Gilmore, Kenneth Elder, Khalida Suri, Kimberley Bossi, Kiran Majid, Kyrllos Farag, Laura Arthur, Lauren Hackney, Lilia Ragad, Livia Walsh, Loaie Maraqa, Louise Alder, Lucy Gossling, Marina Verebcean, Marta D'Auria, Michael Devine, Michael Flanagan, Michael Jones, Michael Kelly, Monica Reeves, Monika Rezacova, Muhammad Hashmi, Myat Win, Natalie Fairhurst, Natalie Hirst, Nicholas Holford, Nicola Cook, Norah Scally, Noyko Stanilov, Nur Nurmahomed, Olamide Oyende, Olaniyi Olayinka, Qian Chen, Rachel Foster, Rachel Lee, Radhika Merh, Rahi Karmarkar, Raouef Ahmed Bichoo, Rashad Abdelrahman, Rashmi Verma, Rebecca Llewellyn-Bennett, Rishabha Sharma, Ritika Rampal, Róisín Tully, Sabina Rashid, Sabreen Elbakri, Sam Jeffreys, Samantha Muktar, Samuel Baxter, Sarah Gibbins, Shahnaz Qureshi, Sharat Chopra, Shiveta Razdan, Simon Pilgrim, Sreekumar Sundara Rajan, Sumbal Bhatti, Sunita Saha, Syed Noor Hussain Shah, Tabitha Grainger, Tahera Arif, Tamara Kiernan, Tasha Gandamihardja, Thalia Picton-Scott, Thomas Hubbard, Titus Murphy, Tom Seddon, Tomasz Graja, Trisha Kanani, Urvashi Jain, Verda Amin, Vijay Narbad, Zoe Barber, Zoe Chia

**Affiliations:** Department of Surgery & Cancer, Imperial College London, London, UK; Imperial College Healthcare NHS Trust, London, UK; King’s Mill Hospital, Sutton-in-Ashfield, UK; Hull University Teaching Hospitals, Hull, UK; Imperial College Healthcare NHS Trust, London, UK; Royal Marsden NHS Foundation Trust, London, UK; Manchester University NHS Foundation Trust, Manchester Academic Health Sciences Centre, Manchester, UK; Singleton Hospital, Swansea, UK; Wythenshawe Hospital, Wythenshawe, Manchester, UK; Imperial College Healthcare NHS Trust, London, UK; Manchester University NHS Foundation Trust, Manchester Academic Health Sciences Centre, Manchester, UK; The Kennedy Institute of Rheumatology Oxford University, Oxford, UK; Imperial College Healthcare NHS Trust, London, UK; Swansea University, Swansea, UK; Belfast Health and Social Care Trust, Belfast, UK; The Royal Devon and Exeter NHS Foundation Trust, Exeter, UK; Southern Health & Social Care Trust, Portadown, UK; NHS Forth Valley, Larbert, UK; Bon Secours Hospital, Cork, Ireland; UCLH, UCL Cancer Institute, London, UK; Patient Representative, London, UK; University of Bristol, Bristol, UK; St Vincent’s University Hospital, Dublin, Ireland; Imperial College Healthcare NHS Trust, London, UK; Imperial College Healthcare NHS Trust, London, UK; Imperial College Healthcare NHS Trust, London, UK; Belfast City Hospital, Belfast Health & Social Care Trust, Belfast, UK; Imperial College Healthcare NHS Trust, London, UK; St Vincent’s University Hospital, Dublin, Ireland; Department of Surgery & Cancer, Imperial College London, London, UK; Imperial College Healthcare NHS Trust, London, UK

## Abstract

**Background:**

The aim of this multicentre prospective audit was to describe the current practice in the management of mastitis and breast abscesses in the UK and Ireland, with a specific focus on rates of surgical intervention.

**Methods:**

This audit was conducted in two phases from August 2020 to August 2021; a phase 1 practice survey and a phase 2 prospective audit. Primary outcome measurements for phase 2 included patient management pathway characteristics and treatment type (medical/radiological/surgical).

**Results:**

A total of 69 hospitals participated in phase 2 (1312 patients). The key findings were a high overall rate of incision and drainage (21.0 per cent) and a lower than anticipated proportion of ultrasound-guided aspiration of breast abscesses (61.0 per cent). Significant variations were observed regarding the rate of incision and drainage (range 0–100 per cent; *P* < 0.001) and the rate of needle aspiration (range 12.5–100 per cent; *P* < 0.001) between individual units. Overall, 22.5 per cent of patients were admitted for inpatient treatment, out of whom which 72.9 per cent were commenced on intravenous antibiotics. The odds of undergoing incision and drainage for a breast abscess or being admitted for inpatient treatment were significantly higher if patients presented at the weekend compared with a weekday (*P* ≤ 0.023). Breast specialists reviewed 40.9 per cent of all patients directly, despite the majority of patients (74.2 per cent) presenting within working hours on weekdays.

**Conclusions:**

Variation in practice exists in the management of mastitis and breast abscesses, with high rates of incision and drainage in certain regions of the UK. There is an urgent need for a national best-practice toolbox to minimize practice variation and standardize patient care.

## Teaser

The Mastitis and Mammary Abscess Management Audit (MAMMA) is the first international audit, conducted in 69 hospitals across the UK and Ireland, involving contemporaneous data capture on the presentation and management of 1312 patients with mastitis and breast abscess. Key results included a substantial inpatient admission rate (22.5 per cent), a high overall rate of surgical incision and drainage (21.0 per cent), and a comparatively low use of ultrasound-guided aspiration (61.0 per cent). The data also identified significantly increased odds of undergoing incision and drainage for a breast abscess or being admitted for inpatient treatment, if patients presented at the weekend compared with on a weekday.

## Introduction

Mastitis and breast abscesses are common benign breast conditions affecting between 5.0 and 33.0 per cent of women, depending on aetiology^1–5^. Currently, there are no published guidelines for the management of mastitis and breast abscesses in the UK and Ireland. Clinical practice is based on international guidelines and local protocols, many of which are outdated^1,2,5,6^. The standard management depends on the aetiology and ordinarily involves antimicrobial treatment, in addition to ultrasound-guided needle aspiration or surgical incision and drainage. Ultrasound-guided needle aspiration is considered to be the standard treatment for breast abscesses^1,2,6,7^, despite the lack of definitive evidence^8^. There are no evidence-based criteria for the selection of patients for surgical incision and drainage.

A recent national practice review by the Getting It Right First Time (‘GIRFT’) initiative identified that breast infections account for 2.5 per cent of all admissions and 1.2 per cent (ranging from 0.3 to 5.0 per cent) of all ‘breast excisions’, representing approximately 2000 women and 1700 potentially avoidable admissions per year^9^. Indeed, anecdotal evidence suggests variation in practice at a local level^7,10,11^, particularly concerning lack of uniformity in first-line antibiotic prescribing, justification for hospital admission, access to ultrasound-guided aspiration, rates of surgical incision and drainage, and duration of inpatient treatment. At least 40 per cent of women are prescribed inappropriate antibiotics^7,11^ and one in three women are admitted for inpatient treatment^7^. The rate of incision and drainage is of particular concern as the incidence differs dramatically between studies, from 0.7 to over 86.5 per cent^7,12^, and surgical intervention is associated with adverse aesthetic and quality-of-life outcomes^13^.

In many centres, breast surgeons are no longer participating in on-call rotas, meaning acute breast infections may be managed by non-specialists out of hours. It is hypothesized that variation in practice exists across the UK and Ireland, especially with respect to hospital admission and incision and drainage rates. The aim of the Mastitis and Mammary Abscess Management Audit (MAMMA) was to evaluate national practice variation in the management of mastitis and breast abscesses in the UK and Ireland, and to provide recommendations for best practice.

## Methods

### Design, setting, and data sources

A multicentre, multi-phase, prospective audit was conducted across the UK and Ireland within secondary care (1 August 2020 to 31 August 2021). All hospitals treating mastitis and breast abscesses were eligible to participate.

The aim of phase 1 was to investigate patient care pathways and sub-specialty involvement in the management of mastitis and breast abscesses. A practice survey was disseminated to local trainee leads and completed in collaboration with the lead supervising consultants on REDCap. REDCap (Research Electronic Data Capture) is a web-based platform used for secure data collection for research studies and audits worldwide, and for the purpose of this project hosted at the The Kennedy Institute of Rheumatology Oxford University.

The aim of phase 2 was to evaluate the management of patients with mastitis and breast abscesses through real-time data capture. To ensure that all suitable patients were identified and included in the data collection, local trainee collaboratives were requested, on a daily basis, to check all presentations to the surgical assessment unit and emergency department (ED), review handover sheets and ward lists, collate new referrals to the breast clinic from the breast team, and liaise with the on-call surgical teams. Contemporaneous data, excluding identifiable information, were collected for all patients from medical records and captured in a REDCap database to avoid data loss. Each patient was given a unique study identification number to avoid accidental data duplication. At the end of phase 2, all data sets were checked for data accuracy and missing data. For data validation, the investigators had to review and verify all data collected for 10 randomly chosen patients.

Regions were defined according to Health Education England deaneries, which resulted in the following division: Ireland, Wales, Scotland, Northern Ireland, and 12 regions across England. To ensure anonymity, regions were allocated a number without a particular order. Two regions in England did not participate in this audit.

### Participants

All female patients over 16 years old presenting with symptoms of mastitis or breast abscess were included. Exclusion criteria included male patients, biopsy-proven breast cancer, breast surgery within 90 days of presentation, and/or breast implant *in situ* on the affected side.

### Outcomes

Data collection proformas for both study phases can be found in the *Supplementary Appendixes*. Briefly, outcomes collected in phase 2 covered five areas, specifically: patient demographics, patient treatment pathway, diagnosis, risk factors, and treatment. Patient demographics included age (years), BMI, and number of weeks postpartum. Patient treatment pathway data included day and time of presentation, grade of admitting doctor, source of referral, use of antibiotics in the community before presentation, and involvement of the breast team. Data were collected on the subtype of mastitis (for example lactational, periductal, etc.) and delay in seeking help from onset of symptoms (days). Information was recorded on risk factors, including breastfeeding, use of breast pumps, previous history of breast infection, and factors not associated with lactation, such as smoking, diabetes, and breast trauma.

Data on treatment(s) included type, duration, route and justification of antibiotic choice, location of treatment (that is primary care outpatients or admitted for secondary care), and, if admitted, documented justification for admission, grade of decision maker, and the duration of hospital stay (days). Access to breastfeeding advice was recorded. Regarding radiological imaging, the use of diagnostic ultrasonography and rate of needle aspiration (with or without ultrasonography guidance, waiting time, and number of aspirations) were recorded. Justification for surgical incision and drainage was documented along with waiting times from decision to operate to surgery (days). Microbiology results were recorded, including specific details on microorganisms cultured.

### Study size

There are 167 breast surgery units in the UK. The aim of this audit was to recruit at least one-third of breast surgery units across the UK and Ireland (56 hospitals). This level of participation was achieved by a number of other national breast surgery projects^14–16^. The interim analysis of phase 1 audit data suggested that the mean number of patients presenting with mastitis or breast abscess per unit per month was around seven, which across 56 units over 3 months would be equivalent to 1176 patient records. To reduce selection bias, participating centres collected data on all consecutive patients who met the inclusion criteria for an interval of 3 months or longer until the minimum number of 21 patients was reached. Where units were unable to reach the required minimum number of patients, despite continuing data collection for 6 months, their data were included in the audit under a proviso of being a small unit.

### Patient and public involvement

A patient representative was recruited to be part of the steering committee and participated in the initial audit design, data analysis, and preparation of the results for publication. Information regarding the audit was available to the public on the http://mammastudy.com/ website.

### Ethical approval

Ethical approval was not required as this study was regarded as an audit by the Medical Research Council (‘MRC’) Health Research Authority (‘HRA’) decision tool. The participation of each hospital site was contingent on obtaining local registration and approval from its clinical governance department, which was followed by issuing of REDCap access and commencement of data collection.

### Statistical methods

Statistical analysis was performed using open-source software JASP Team (2020)^17^, results of which have been extensively verified against other popular statistical software^18^. Funnel plots were generated using RStudio^19^. Descriptive statistics were used to summarize frequencies of observed values. All numerical data were checked for normal distribution (Q–Q plots) and homogeneity of variance (Levene’s test). ANOVA was used for independent parametric data. Where measures were non-parametric, the Kruskal–Wallis test was employed. The chi-squared test was used to compare categorical data. ORs were calculated to determine the relative odds of the occurrence of the outcomes. Logistic regression analysis was used to examine associations. A *P* value of <0.050 was considered significant for all statistical tests.

## Results

### Demographics and risk factors

Results of phase 1 are presented in the *Supplementary Results*. A total of 69 hospitals participated in phase 2 (England, 55; Scotland, 3; Wales, 3; Northern Ireland, 2; and Ireland, 6). Data were entered for 1350 patients, of which 38 records were excluded due to being incomplete (11 records), age <16 years old (10 records), no diagnosis of mastitis or abscess (12 records), and/or missing data in the diagnosis field (5 records).

The median patient age was 36 (interquartile range (i.q.r.) 31–46) years. A significant difference (*P* < 0.001) was observed in the age of patients diagnosed with lactational and non-lactational disease (median age of 33 (i.q.r. 29–35) years for patients diagnosed with lactational disease and 40 (i.q.r. 32–51) years for patients diagnosed with non-lactational disease) (*Fig. S1*). Over one-third of patients were obese (BMI greater than 30 kg/m^2^) (*Fig. S2*). The median time from delivery to developing lactational mastitis (201 instances) was 6 (i.q.r. 3–15) weeks and for lactational breast abscesses (193 instances) the median time was also 6 (i.q.r. 4–9) weeks.

Risk factors varied significantly depending on the type of mastitis or breast abscess (*Figs S3*, *S4*). Smoking was an independent predictor of periductal mastitis and abscess (*P* < 0.001), as well as peripheral mastitis and abscess (*P* < 0.001).

### Treatment pathway

**Fig. 1:**
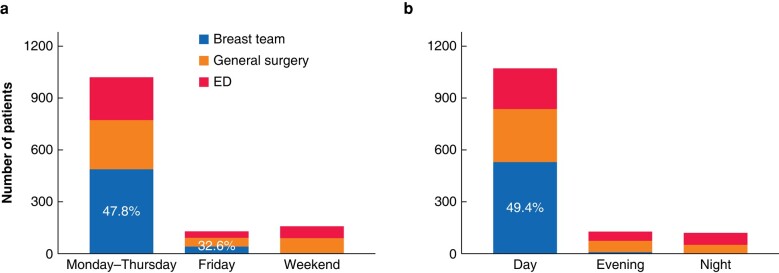
Relationship between the a) day of presentation and b) time of presentation and initial specialty involvement

Most patients presented on weekdays (Monday to Thursday) during normal working hours (*Fig. S5* and *Table S1*). Specialty involvement varied depending on the timing of presentation, with breast teams being available for direct referral between Monday and Friday during normal working hours (*Fig. 1*). Less than half of all patients were reviewed by the breast team, even during normal working hours between Monday and Thursday. Although the proportion of patients presenting on Friday and at weekends was smaller, most were reviewed by the on-call general surgery or ED team first.

General practitioners (GPs) were the predominant source of referral between Monday and Friday during normal working hours, whereas referrals were primarily from EDs out of hours. Referrals from maternity services and direct self-referral were rare (*Fig. S6*). Of those referred by EDs, 216 (49.4 per cent) were reviewed by GPs before ED attendance. Patients with breast abscesses were significantly more likely to see their GP before ED presentation compared with patients with mastitis (OR 2.52, 95 per cent c.i. 1.69 to 3.76; χ^2^ = 21.1; *P* < 0.001) (*Table 1*).

**Table 1:** Characteristics of patients, depending on the type of diagnosis

Diagnosis	Seen by GP before ED presentation	Breast team follow-up	Inpatient treatment	Antibiotics in primary care	Antibiotics in secondary care	Needle aspiration	Incision and drainage
Lactational mastitis	33 (16.4)	133 (66.2)	72 (35.8)	120 (59.7)	154 (76.6)	8 (4.0)	1 (0.5)
Lactational breast abscess	42 (21.8)	168 (87)	50 (25.9)	151 (78.2)	174 (90.2)	157 (81.3)	35 (18.1)
Periductal mastitis	12 (9.1)	97 (73.5)	9 (6.8)	75 (56.8)	97 (73.5)	16 (12.1)	1 (0.8)
Periductal breast abscess	47 (19.4)	212 (87.6)	45 (18.6)	178 (73.6)	199 (82.2)	144 (59.5)	42 (17.4)
Peripheral non-lactational mastitis	17 (9.3)	137 (74.5)	42 (23.0)	107 (58.5)	140 (76.4)	36 (19.7)	8 (4.4)
Peripheral non-lactational breast abscess	64 (19.1)	271 (80.9)	72 (21.5)	236 (70.4)	271 (80.9)	169 (50.4)	85 (25.4)
Granulomatous mastitis	1 (3.8)	21 (80.8)	5 (19.2)	15 (57.7)	20 (76.9)	13 (50.0)	3 (11.5)

Values are *n* (%). ED, emergency department.

Patients waited for a median of 5 (i.q.r. 3–10) days from onset of symptoms to seeking medical help. The majority (1179 patients, 89.9 per cent) were assessed by the breast team at some stage during their treatment pathway in the outpatient setting (972 patients, 82.4 per cent) and/or as an inpatient (202 patients, 17.1 per cent). Breast clinic follow-up was arranged for 1039 (79.2 per cent) patients. Patients with abscesses were more likely to be followed up in the breast clinic (OR 3.43, 95 per cent c.i. 2.35 to 5.01; χ^2^ = 44.5; *P* < 0.001) (*Table 1*). There was a significant variation in the pooled regional rate of the specialist breast team follow-up (χ^2^ = 58.9; *P* = 0.002).

### Treatment

#### Medical

##### Inpatient treatment

Advice to continue breastfeeding was given to 296 (80.7 per cent) lactating women. Over three-quarters of patients were treated in the outpatient setting (1015 patients, 77.4 per cent); 295 patients (22.5 per cent) were admitted to hospital for a median of 2 (i.q.r. 2–4) days. The likelihood of being admitted depended on the diagnosis (*Table 1*). Reasons for admission are listed in *Table 2*. The decision to admit was made predominantly by specialist registrars (174 patients, 59.0 per cent) regardless of the time or day of presentation (*Fig. S7*). The odds of admission were significantly higher at the weekend compared with on Monday and Friday (OR 2.68, 95 per cent c.i. 1.89 to 3.80; χ^2^ = 32.6; *P* < 0.001). There was a significant variation between individual centres regarding the rate of inpatient treatment (χ^2^ = 291.8; *P* < 0.001). Certain regions admitted about one-third of presenting patients for inpatient treatment.

**Table 2:** Admission and treatment-related statistics

**Reasons for admission**	
Intravenous antibiotics	215 (72.9)
Severe infection/sepsis	121 (41.0)
Rapidly progressing infection	18 (6.1)
Haemodynamic instability	5 (1.7)
Immunocompromised	2 (0.7)
**Reasons for incision and drainage**	
Skin changes or necrosis	79 (45.1)
Pointing	61 (34.9)
Size ≥5 cm	30 (17.1)
Multiloculated abscess	27 (15.4)
Duration of symptoms ≥5 days	64 (36.6)
Other	23 (13.1)
**Factors associated with increased odds of incision and drainage, OR (95% c.i.), χ^2^, *P***	
Co-morbidities	2.0.4 (1.35,3.09), 11.7, <0.001
Severe infection/sepsis	3.36 (2.19,5.13), 34.2, <0.001
Rapidly progressing infection	13.85 (5.12,37.41), 44.8, <0.001
Use of antibiotics in the community	1.53 (1.07,2.20), 5.3, 0.021

Values are *n* (%) unless otherwise indicated.

##### Antibiotics

Before presenting to secondary care, 882 (67.2 per cent) patients were treated with antibiotics, of which the majority (679 patients, 77.0 per cent) had one antibiotic course (*Table 3*). Oral antibiotics administered in primary care significantly reduced the need for intravenous antibiotics in secondary care (OR = 0.71, 95 per cent c.i. 0.54 to 0.94; χ^2^ = 5.6; *P* = 0.018). Of patients seen in secondary care, 1055 (80.4 per cent) were started on antibiotics, of which 767 (72.7 per cent) were started on oral antibiotics. Co-amoxiclav and flucloxacillin were the first-line antibiotics of choice (*Fig. S8*). Patients with abscesses were significantly more likely to be commenced on antibiotics compared with patients with mastitis both in primary care (OR 1.97, 95 per cent c.i. 1.55 to 2.48; χ^2^ = 32.4; *P* < 0.001) and secondary care (OR 1.64, 95 per cent c.i. 1.25 to 2.16; χ^2^ = 12.7; *P* < 0.001) (*Table 1*).

**Table 3:** Antibiotics in primary and secondary care

	Primary-care antibiotics	Secondary-care antibiotics				
		Overall	Primary-care antibiotics		Route	
			No	Yes	Oral	Intravenous
**Antibiotic course**						
First	679 (77.0)	–	300 (69.9)	506 (57.4)	767 (72.7)	277 (26.3)
Second	145 (16.6)	–	56 (13.1)	135 (15.3)	136 (12.9)	53 (5.0)
Third or above	47 (5.3)	–	11 (2.6)	22 (2.5)	26 (2.5)	7 (0.7)
**Course length**						
<7 days	–	–	–	–	–	165 (59.6)
7–10 days	–	–	–	–	–	83 (30.0)
11–14 days	–	–	–	–	–	22 (7.9)
>14 days	–	–	–	–	–	5 (1.8)
**First-line antibiotic (top three)**						
Co-amoxiclav	–	450 (42.7)	–	–	–	–
Flucloxacillin	–	389 (36.9)	–	–	–	–
Clindamycin	–	84 (8.0)	–	–	–	–
**Course length of the first-line antibiotic**						
<7 days	–	368 (34.9)	–	–	–	–
7–10 days	–	485 (46.0)	–	–	–	–
11–14 days	–	131 (12.4)	–	–	–	–
>14 days	–	54 (5.1)	–	–	–	–
**Reason for selection of the first-line antibiotic (top three)**						
Local protocol	–	714 (67.7)	–	–	–	–
Prior treatment	–	131 (12.4)	–	–	–	–
Drug allergies	–	90 (8.5)	–	–	–	–
**Second-line antibiotic (top three)**						
Co-amoxiclav	–	40 (22.5)	–	–	–	–
Flucloxacillin	–	43 (24.2)	–	–	–	–
Metronidazole	–	64 (36)	–	–	–	–

Values are *n* (%).

#### Radiology

A diagnostic breast ultrasonography scan was performed for 1061 (80.9 per cent) patients, with significant variation regarding the rate of ultrasonography scans performed depending on the diagnosis (χ^2^ = 21.1; *P* = 0.002). All patients with granulomatous mastitis had a diagnostic breast ultrasonography scan. The median waiting time to obtain a breast ultrasonography scan was 1 (i.q.r. 0–2) day. The rate of needle aspiration was the highest for patients with lactational breast abscesses. Overall, 453 (88.5 per cent) needle aspirations were performed under ultrasonography guidance on the same day (i.q.r. 0–1); most patients required 1 aspiration (i.q.r. 1–2). There was a significant variation between individual centres regarding the rate of diagnostic breast ultrasonography scans performed (χ^2^ = 254.1; *P* < 0.001) for all presentations and needle aspiration of breast abscess (χ^2^ = 179.4; *P* < 0.001), ranging from 12.5 to 100 per cent.

#### Surgery

**Fig. 2:**
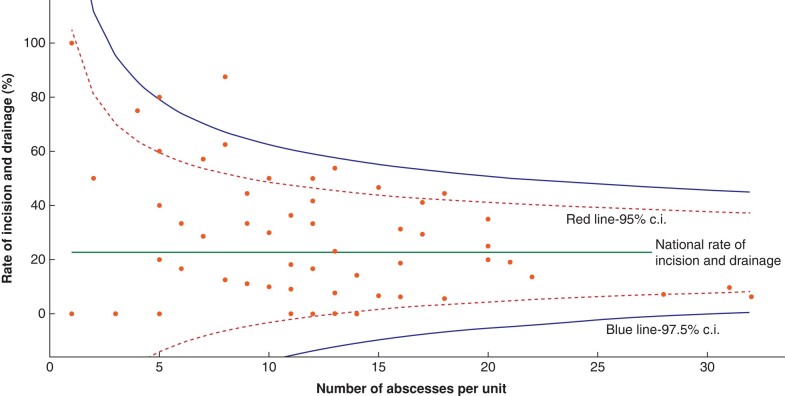
Individual centre rate of incision and drainage, and the number of abscesses seen during the audit data collection interval (typically 3 months)

Overall, the surgical incision and drainage rate varied between 17.4 and 25.4 per cent depending on the abscess aetiology (*Table 1*). The median waiting time for incision and drainage was 1 (i.q.r. 0–3) day. Only 2.9 per cent (five instances) required repeat operation. Justifications for surgical incision and drainage are listed in *Table 2*. The odds of undergoing incision and drainage for a breast abscess were significantly higher in patients presenting at weekends compared with patients presenting on weekdays (OR 1.76, 95 per cent c.i. 1.08 to 2.86; χ^2^ = 5.2; *P* = 0.023). There was a significant variation in the surgical incision and drainage rate between individual centres (χ^2^ = 153.3; *P* < 0.001) (*Fig. 2*), ranging from 0 to 100 per cent.

Receiving surgical incision and drainage significantly increased the odds of admission compared with patients who were treated conservatively (OR 1.62, 95 per cent c.i. 1.24 to 2.00; χ^2^ = 77.3; *P* < 0.001). There was a significant association between the need for surgical incision and drainage, and the requirement for intravenous antibiotics (OR 2.10, 95 per cent c.i. 1.46 to 3.03; χ^2^ = 16.6; *P* < 0.001), as well as the prescription of a short course of antibiotics (<7 days) within the hospital setting (χ^2^ = 12.0; *P* = 0.007). Needle aspiration significantly reduced the risk of surgical incision and drainage (OR 0.31, 95 per cent c.i. 0.22 to 0.45; χ^2^ = 43.0; *P* < 0.001). Factors associated with an increased odds of surgical incision and drainage are listed in *Table 2*.

#### Microbiology

Pus was sent for culture and sensitivity in 503 (92.6 per cent) cases of needle aspirations and 155 (88.6 per cent) cases of surgical incision and drainage. Overall, a pathogenic organism was successfully isolated from 349 (64.3 per cent) aspiration samples and 99 (56.6 per cent) surgical incision and drainage samples. *Staphylococcus* species were the most frequently isolated organisms (253 instances).

## Discussion

The MAMMA is the first multicentre audit covering the UK and Ireland to confirm high rates of surgical incision and drainage, inpatient admission, and loss to follow-up amongst patients presenting with mastitis and breast abscesses.

Despite a worldwide drive to reduce the rate of surgical incision and drainage for breast abscesses through the use of needle aspiration over the last two decades^7,20–29^, findings from the MAMMA suggest the persistent use of incision and drainage (ranging from 0 to 100 per cent of patients, depending on the centre). Surgical incision and drainage is complicated by interference with lactation, prolonged wound healing^26,28^, risk of mammary-duct fistulae^30^, and dissatisfaction with poor cosmetic outcomes^13^. Furthermore, surgical intervention leads to additional healthcare-provider costs, including the need for general anaesthesia, inpatient recovery, nursing care, and wound care^11^.

Over one-third of patients underwent aspiration before surgical incision and drainage; all patients had a documented indication. Significant regional variation in surgical incision and drainage rates, however, suggests that there may be scope to reduce operative intervention. The most frequent justifications for incision and drainage were skin changes, necrosis, pointing, and duration of symptoms over 5 days, for which there is no established evidence base. Indications for which there does exist a weak evidence base are abscess size over 5 cm and multiloculation^26,31^; however, these were less frequently observed indications for incision and drainage. Critically, none of the current guidelines^1,2,5,6^ specifies indications for surgical incision and drainage. The view is unless there is skin necrosis or at least one failed attempt at ultrasound-guided aspiration, patients should not proceed to surgery. Arguably, even for multiloculation, there is evidence that such abscesses can be successfully treated with a vacuum biopsy system, negating the need for surgical incision and drainage ^32^.

There was an association between rates of surgical incision and drainage and inpatient treatment. Almost three-quarters of patients undergoing incision and drainage were admitted for intravenous antibiotics. The average cost of an inpatient bed is around £350 per day. If an intervention such as surgical incision and drainage is performed, then the cost rises to over 2880 EUR per admission^33^. The cost of an outpatient ultrasound-guided aspiration by comparison is on average 76 EUR^33^. Assuming that two-thirds of the recorded surgical incision and drainage procedures could have been successfully treated non-operatively through image-guided aspiration, this could represent a saving of at least 1.15 EUR million per annum for participating centres. Reducing rates of surgical incision and drainage is therefore desirable to reduce associated healthcare costs.

Undergoing surgical incision and drainage, and being admitted to hospital were significantly more likely if patients presented at the weekend. This is likely to be multifactorial in nature and at least partially explained by the lack of access to ultrasound-guided aspiration at the weekend. At present, the MAMMA identified that only 40 per cent of patients are seen directly by breast surgeons during normal working hours. As breast surgical trainees enter a more dedicated training pathway^34^, there is a great opportunity for such trainees to become more skilled in managing emergent breast conditions, which will only serve to improve patient care. Accreditation in breast ultrasonography for surgeons^35,36^, for example, could be incorporated in the new training pathway and be utilized out of hours to minimize the need for surgical incision and drainage, whilst also alleviating pressure on breast radiologists, where there is a current 29 per cent shortfall^37^. Additionally, there may be an opportunity to involve the extended surgical team, by training nurse practitioners and extended-role radiographers to perform aspirations and to manage these patients^38^.

In the more immediate term, the patient’s journey could be reshaped. Over 90 per cent of referrals are generated by GPs and EDs, yet only one-quarter of hospitals provide a direct pathway from these referrers to the breast clinic, with the majority requiring prior assessment by the on-call general surgery team. Establishing direct and rapid access to the breast clinic and breast imaging would ensure prompt and appropriate patient care and reduce resource wastage. Another option could be to allow patient self-referral. There is a growing evidence base in other specialties that provision of such a service improves resource use, reduces ED visits, and reduces inappropriate admissions^39–42^.

An alternative solution could be to train front-line ED staff and emergency general surgeons to perform needle aspiration with or without ultrasonography guidance. This could be used as a temporizing measure out of hours until the patient is seen in the breast clinic. Many ED staff are already competent in performing bedside ultrasonography, particularly those trained in Focused Assessment with Sonography for Trauma (‘FAST’) scanning. Emergency aspiration may not completely resolve the abscess, but it could reduce the risk of worsening infection, skin changes, and necrosis, as well as alleviate the patient’s discomfort. For this pathway to be effective one needs to consider the high turnover of staff^7^, particularly in the current climate of staff shortages and prolonged waiting times in EDs^43,44^.

Several limitations of this audit must be acknowledged. The current data set did not record the specialty performing surgical incision and drainage; specifically it is unknown if emergency general surgeons or breast surgeons performed procedures. This limits the ability to extrapolate the extent to which the service can be improved if it was delivered entirely by breast surgeons. The proforma did not allow selection of surgical incision and drainage as the sole reason for admission, making it difficult to establish causality between admission for inpatient treatment, intravenous antibiotics, and operative intervention. Regarding regional variation, 10 units (14.7 per cent) documented less than 10 patient cases, potentially skewing results. Although the data represent the UK and Ireland population only, the results are applicable internationally, considering the wide variation in reported rates of surgical incision and drainage^7,12^. The recommendations proposed here align well with the recent US guidelines^6^. Furthermore, reducing the rate of surgical incision and drainage and inpatient admissions, and increasing the rate of needle aspirations are more cost-effective measures, which are of greater relevance in developing countries, where resources are limited.

Ultimately, achieving improvements and standardization of patient care is not feasible without an updated set of national and international guidelines, implementation of changes at a local level, and ongoing re-auditing of outcomes. There is an urgent need to re-imagine patient care pathways to reduce surgical incision and drainage rates, minimize unnecessary hospital admissions, and maximize the use of resources.

## Collaborators

**MAMMA Research Collaborative**

Ahmed Ahmed (Royal Derby Hospital, Derby, UK); Ahmed Shalaby (Buckingham Healthcare Trust, High Wycombe, UK); Akanksha Kiran (Dorset County Hospital Foundation Trust, Dorchester, UK); Alexander Boucher (King's Mill Hospital, Sutton-in-Ashfield, UK); Alexander Ribbits (Conquest Hospital, Hastings, UK); Alexandra Tenovici (Wexham Park Hospital, Slough, UK); Alice Chambers (North Bristol NHS Trust, Bristol, UK); Alice Lee (West Middlesex University Hospital, Isleworth, UK); Alison Bate (Torbay and South Devon NHS Foundation Trust, Torquay, UK); Amanda Koh (Nottingham University Hospital NHS Trust, Nottingham, UK); Anita Sharma (Torbay and South Devon NHS Foundation Trust, Torquay, UK); Anjelli Wignakumar (Colchester General Hospital, Colchester, UK); Anna Fullard (University Hospital Galway, Galway, Ireland); Anna Isaac (Belfast City Hospital, Belfast, UK); Anneliese Lawn (Ashford and St Peter's Hospital, Chertsey, UK); Aonghus Ansari (Leicester Glenfield Hospital, Leicester, UK); Arjuna Brodie (Leicester Glenfield Hospital, Leicester, UK); Arthika Surendran (University Hospital Coventry & Warwickshire, Coventry, UK); Ashvina Segaran (Thames Valley HEE, Oxford, UK); Ayesha Abbasi (Southend University Hospital, Southend-on-Sea, UK); Azel Regan (Nevill Hall Hospital, Abergavenny, UK); Badr Al-Khazaali (Princess Royal Hospital, Orpington, UK); Bahar Mirshekar-Syahkal (West Suffolk Hospital, Bury St Edmunds, UK); Bahaty Riogi (St Helens & Knowsley Teaching Hospitals NHS Trust, Prescot, UK); Benjamin Patel (North Bristol NHS Trust, Bristol, UK); Brenda Muntean (Queens Hospital Burton, London, UK); Buket Ertansel (St George's Hospital, London, UK); Candice Downey (Airedale General Hospital, Keighley, UK); Carolyn Cullinane (St Vincent's University Hospital, Dublin, Ireland); Catherine Rossborough (Craigavon Area Hospital, Craigavon, UK); Charlotte Kallaway (Frimley Park Hospital, Frimley, UK); Chiara Sirianni (Betsi Cadwaleadr UHB West, Bangor, UK); Chwanrow Baban (Wexham Park Hospital, Slough, UK); Ciaran Hollywood (Chesterfield Royal Hospital, Chesterfield, UK); Clare Roger (Doncaster & Bassetlaw Teaching Hospitals, Doncaster, UK); Colin McIlmunn (Belfast City Hospital, Belfast, UK); Deeksha Arora (Royal Derby Hospital, Derby, UK); Despoina Chatzopoulou (Frimley Park Hospital, Frimley, UK); Diya Mirghani (Cumberland Infirmary Carlisle, Carlisle, UK); Ed Babu (Hillingdon Hospital, Uxbridge, UK); Eilidh Bruce (Aberdeen Royal Infirmary, Aberdeen, UK); Eiman Khalifa (Castle Hill Hospital, Cottingham, UK); Elaf Osman (University Hospital Waterford, Waterford, Ireland); Eleftheria Kleidi (Cambridge University Hospitals, Cambridge, UK); Eleni Ntakomyti (University College London Hospital, London, UK); Emma Kellett (Hull University Teaching Hospitals, Hull, UK); Erum Najeeb (Derriford Hospital, Plymouth, UK); Evangelos Mallidis (Ipswich Hospital, Ipswich, UK); Fiona Rutherford (Royal Alexandra Hospital, Brighton, UK); Francesca Malcolm (Chesterfield Royal Hospital, Chesterfield, UK); Francesk Mulita (General University Hospital of Patras, Rio, Greece); Gabriella Marchitelli (Royal Victoria Infirmary, Newcastle upon Tyne, UK); Gemma Hughes (Northampton General Hospitals, Northampton, UK); George Neelankavil Davis (Dorset Couny Hospital, Dorchester, UK); Georgios Karagiannidis (Ipswich Hospital, Ipswich, UK); Ghadah Alyahya (Leighton hospital, Crewe, UK); Ghassan Elamin (King's Mill Hospital, Sutton-in-Ashfield, UK); Giovanni Santoro (Countess of Chester Hospital NHS Trust, Chester, UK); Goran Ahmed (Frimley Park Hospital, Frimley, UK); Grace Knudsen (Frimley Park Hospital, Frimley, UK); Grant Harris (Northumbria NHS Foundation Trust, Newcastle upon Tyne, UK); Gwen Bromley (Queen Elizabeth Hospital, London, UK); Hana Esack (Lincoln County Hospital, Lincoln, UK); Hannah Markey (University Hospital Galway, Galway, Ireland); Harry Yeuk Hei Lei (Charing Cross Hospital, London, UK); Heather Pringle (Royal Devon and Exeter Hospital, Exeter, UK); Hedwige Nathaniel (Northwick Park Hospital, Harrow, UK); Henry D Robb (Charing Cross Hospital, London, UK); Hytham K. S. Hamid (William Harvey Hospital, Ashford, UK); Ibrahim Elzayat (Poole Hospital, Poole, UK); Ishita Handa (University Hospital Southampton, Southampton, UK); Jaideep Rait (Maidstone & Tunbridge Wells NHS Trust, Maidstone, UK); Javeria Iqbal (Leicester Glenfield Hospital, Leicester, UK); Jayan George (Sheffield Teaching Hospitals NHS Foundation Trust, Sheffield, UK); Jenna Morgan (Doncaster Royal Infirmary, Doncaster, UK); Jennifer Long (Royal Glamorgan Hospital, Pontyclun, UK); Jenny Banks (Torbay and South Devon NHS Foundation Trust, Torquay , UK); Jih Dar Yau (Hull University Teaching Hospitals, Hull, UK); Joanna Stringer (Northampton General Hospital, Northampton, UK); Joey Fong (St Helens & Knowsley Teaching Hospitals NHS Trust, Prescot, UK); Joseph Maalo (West Hertfordshire Teaching Hospitals NHS Trust, St Albans, UK); Josh Marston (Hull University Teaching Hospitals, Hull, UK); Joshua Silva (Charing Cross Hospital, London, UK); Julia Massey (Chesterfield Royal Hospital, Chesterfield, UK); Katharine Kirkpatrick (Bedfordshire Hospitals, Bedford, UK); Katherine De Rome (West Middlesex University Hospital, Isleworth, UK); Katherine Fairhurst (Royal United Hospital Bath, Bath, UK); Katie Campbell (Wythenshawe Hospital, Manchester, UK); Katie Gilmore (Royal United Hospital Bath, Bath, UK); Kenneth Elder (Western General Hospital, Edinburgh, UK); Khalida Suri (University College London Hospital, London, UK); Kimberley Bossi (Frimley Park Hospital, Frimley, UK); Kiran Majid (Royal Derby Hospital, Derby, UK); Kyrllos Farag (West Suffolk Hospital, Bury St Edmunds, UK); Laura Arthur (Royal Alexandra Hospital, Brighton, UK); Lauren Hackney (Belfast City Hospital, Belfast, UK); Lilia Ragad (Princess Royal University Hospital, Orpington, UK); Livia Walsh (Ashford and St Peter's Hospitals NHS Foundation Trust, Chertsey, UK); Loaie Maraqa (Royal Hallamshire Hospital, Sheffield, UK); Louise Alder (University Hospital Southampton, Southampton, UK); Lucy Gossling (Royal Derby Hospital, Derby, UK); Marina Verebcean (Diana Princess of Wales Hospital, Grimsby, UK); Marta D'Auria (Lincoln County Hospital, Lincoln, UK); Michael Devine (University Hospital Limerick, Limerick, Ireland); Michael Flanagan (University Hospital Waterford, Waterford, Ireland); Michael Jones (Cheltenham General Hospital, Cheltenham, UK); Michael Kelly (West Middlesex University Hospital, Isleworth, UK); Monica Reeves (St Helens & Knowsley Teaching Hospitals NHS Trust, Prescot, UK); Monika Rezacova (Poole General Hospiral, Poole, UK); Muhammad Hashmi (Dorset County Hospital Foundation Trust, Dorchester, UK); Myat Win (West Hertfordshire Teaching Hospitals NHS Trust, St Albans, UK); Natalie Fairhurst (University College London Hospital, London, UK); Natalie Hirst (Sheffield Teaching Hospitals NHS Foundation Trust, Sheffield, UK); Nicholas Holford (Charing Cross Hospital, London, UK); Nicola Cook (Great Western Hospital, Swindon, UK); Norah Scally (Craigavon Area Hospital, Craigavon, UK); Noyko Stanilov (University College London Hospital, London, UK); Nur Nurmahomed (Chelsea and Westminster Hospital, London, UK); Olamide Oyende (Nottingham University Hospital NHS Trust, Nottingham, UK); Olaniyi Olayinka (Barnsley Hospital NHS Foundation Trust, Barnsley, UK); Qian Chen (Frimley Park Hospital, Frimley, UK); Rachel Foster (Countess of Chester Hospital NHS Trust, Chester, UK); Rachel Lee (Royal Derby Hospital, Derby, UK); Radhika Merh (Maidstone and Tunbridge Wells, Maidstone, UK); Rahi Karmarkar (Princess of Wales Hospital, Bridgend, UK); Raouef Ahmed Bichoo (Hull University Teaching Hospitals, Hull, UK); Rashad Abdelrahman (Lincoln County Hospital, Lincoln, UK); Rashmi Verma (Royal Bolton Hospital, Bolton, UK); Rebecca Llewellyn-Bennett (Cheltenham General Hospital, Cheltenham, UK); Rishabha Sharma (Royal United Hospital Bath, Bath, UK); Ritika Rampal (Hull University Teaching Hospitals, Hull, UK); Róisín Tully (Cork University Hospital, Cork, Ireland); Sabina Rashid (Northwick Park Hospital, Harrow, UK); Sabreen Elbakri (Ninewells hospital, Dundee, UK); Sam Jeffreys (Royal Glamorgan Hospital, Pontyclun, UK); Samantha Muktar (Addenbrooke's Hospital, Cambridge, UK); Samuel Baxter (Queen Elizabeth Hospital, London, UK); Sarah Gibbins (St James's University Hospital, Leeds, UK); Shahnaz Qureshi (Northwick Park Hospital, Harrow, UK); Sharat Chopra (Cardiff and Vale University Health Board, Cardiff, UK); Shiveta Razdan (Wexham Park Hospital, Slough, UK); Simon Pilgrim (Leicester Glenfield Hospital, Leicester, UK); Sreekumar Sundara Rajan (Nottingham University Hospitals NHS Trust, Nottingham, UK); Sumbal Bhatti (Norfolk and Norwich University Hospital, Norwich, UK); Sunita Saha (Colchester General Hospital, Colchester, UK); Syed Noor Hussain Shah (Bon Secours Hospital, Cork, Ireland); Tabitha Grainger (Charing Cross Hospital, London, UK); Tahera Arif (Wirral University Teaching Hospital, Wirral, UK); Tamara Kiernan (St Helens & Knowsley Teaching Hospitals NHS Trust, Prescot, UK); Tasha Gandamihardja (Broomfield Hospital, Chelmsford, UK); Thalia Picton-Scott (St George's Hospital, London, UK); Thomas Hubbard (Royal Devon and Exeter Hospital, Exeter, UK); Titus Murphy (Guy's and St Thomas' NHS Trust, London, UK); Tom Seddon (Kettering Hospital, Kettering, UK); Tomasz Graja (Dorset County Hospital Foundation Trust, Dorchester, UK); Trisha Kanani (Nottingham University Hospital NHS Trust, Nottingham, UK); Urvashi Jain (Guys and St Thomas' NHS Foundation Trust, London, UK); Verda Amin (Warwick Hospital, Warwick, UK); Vijay Narbad (King's Mill Hospital, Sutton-in-Ashfield, UK); Zoe Barber (Princess of Wales Hospital, Bridgend, UK); Zoe Chia (Nottingham University Hospital NHS Trust, Nottingham, UK).

## Supplementary Material

znad333_Supplementary_Data

## Data Availability

Data reported in this paper cannot be found in publicly available databases. Individual centres have access to own data and will have access to the full report.
