# Supplementary material for: Mastitis and Mammary Abscess Management Audit (MAMMA) in the UK and Ireland
Source: Br J Surg. 2023 Oct 31;111(1):znad333. doi: 10.1093/bjs/znad333 (PMC10771135; doi:10.1093/bjs/znad333)
Supplement: znad333_Supplementary_Data [file znad333_supplementary_data.docx]

**Title: MAMMA: Mastitis and Mammary Abscess Management Audit in United Kingdom and Ireland**

Authors: Alona Courtney^1^, Jonathon Clymo^2^, Ruth Parks^3^, Alexander Wilkins^4^, Ruth Brown^2^, Rachel O'Connell^5^, Rajiv Dave^6^, Marianne Dillon^7^, Hiba Fatayer^8^, Rachel Gallimore^2^, Ashu Gandhi^6^, Matthew Gardiner^9^, Victoria Harmer^2^, Lyndsey Hookway^10^, Gareth Irwin^11^, Charlotte Ives^12^, Helen Mathers^13^, Juliette Murray^14^, D. Peter O’Leary^15^, Neill Patani^16^, Sophie Paterson^17^, Shelley Potter^18^, Ruth Prichard^19^, Giovanni Satta^2^, TG Teoh^2^, Paul Ziprin^2^, Stuart McIntosh^20^, Michael R Boland^2,19^, Daniel Richard Leff^1,2^, On behalf of the MAMMA Research Collaborative

^1^Imperial College London, London, England, United Kingdom,

^2^Imperial College Healthcare NHS Trust, London, England, United Kingdom,

^3^King’s Mill Hospital, Sutton-in-Ashfield, England, United Kingdom,

^4^Hull University Teaching Hospitals, England, United Kingdom,

^5^Royal Marsden NHS Foundation Trust, London, England, United Kingdom,

^6^Manchester University NHS Foundation Trust, Manchester Academic Health Sciences Centre, Manchester, England, United Kingdom,

^7^Singleton Hospital, Swansea, Wales, United Kingdom,

^8^Wythenshawe Hospital, Wythenshawe, Manchester, England, United Kingdom,

^9^The Kennedy Institute of Rheumatology Oxford University, Oxford, England, United Kingdom,

^10^Swansea University, Swansea, Wales, United Kingdom,

^11^Belfast Health and Social Care Trust, Belfast, Northern Ireland, United Kingdom,

^12^The Royal Devon and Exeter NHS Foundation Trust, Exeter, England, United Kingdom,

^13^Southern Health & Social Care Trust, Portadown, Northern Ireland, United Kingdom,

^14^NHS Lanarkshire, Scotland, United Kingdom,

^15^Bon Secours Hospital, Cork, Ireland,

^16^UCLH, UCL Cancer Institute, London, England, United Kingdom,

^17^Patient Representative, London, England, United Kingdom,

^18^University of Bristol, Bristol, England, United Kingdom,

^19^St Vincent’s University Hospital, Dublin, Ireland,

^20^Belfast City Hospital, Belfast Health & Social Care Trust, Northern Ireland, United Kingdom

**Corresponding author.**

Alona Courtney MBChB BSc(Hons) MSc MRCS

Imperial College London, Paterson Wing, St Mary's Campus, Praed Street, London, W2 1NY

alona.courtney@icloud.com

**ORCID ID: 0000-0002-0077-1994**

**Supplementary Materials - Index**

| **Supplementary Results** |  |
| --- | --- |
| Phase 1: Practice survey | *pag. 3* |
| **Supplementary Appendixes** |  |
| Data collection for phase 1 | *pag. 4* |
| Data collection for phase 2 | *pag. 6* |
| MAMMA Research Collaborative | *pag. 8* |
| Acknowledgements | *pag. 11* |
| **Supplementary Figures and Tables** |  |
| Table S1. Timing of presentation. | *pag. 12* |
| Figure S1. Median age of patients at the time of diagnosis. | *pag. 13* |
| Figure S2. Body mass index of patients. | *pag. 14* |
| Figure S3. Relationship between the risk factors and the type of mastitis or breast abscess. | *pag. 15* |
| Figure S4. Frequency of the risk factors for development of mastitis and breast abscesses. | *pag. 16* |
| Figure S5. Timing of presentation. | *pag. 17* |
| Figure S6. Relationship between the a) day of presentation and b) time of presentation and the source of referral. | *pag. 18* |
| Figure S7. Relationship between the a) day of presentation and b) time of presentation and the seniority of decision maker. | *pag. 19* |
| Figure S8. Choice of antibiotics in the secondary care. | *pag. 20* |

**Supplementary Results**

## Phase 1: Practice Survey

Eighty-six hospitals submitted a practice survey. A local mastitis and breast abscess protocol was present in 67 (77.9%) hospitals. The predominant location of treatment was assumed to be the breast outpatient clinic (n=69, 80.2%). 75 (87.2%) responding breast units anticipated the breast team to be leading care, rather than the on call general surgery, with 68 (79.1%) of respondents expecting patients to be reviewed by breast specialists on the day of presentation or the following day.

Limited access to breastfeeding support was noted across the majority of responding hospitals. 26 hospitals (30.2%) offered breast feeding support on a post-natal ward, 23 (26.7%) hospitals had access to lactational specialists, 18 (20.9%) hospitals allowed admission of mother and baby and only 21 (24.4%) hospitals provided patient information leaflets.

72 (83.7%) hospitals aimed to review all patients presenting with mastitis and breast abscesses in an outpatient setting (n=79, 91.9%) either on the same day (n=20, 23.5%) or the following day (n=42, 49.4%). Out of hours cover was provided by general surgeons in the majority of hospitals (n=77, 89.5%). Half did not have dedicated appointments in breast clinic for patients with mastitis and breast abscesses; however, 30 (34.9%) hospitals provided daily dedicated emergency clinic appointments. A quarter of hospitals that provided dedicated appointments enabled direct access by emergency department (ED) staff and general practitioners (GPs) and 44 (51.2%) units also permitted general surgeons to book patients directly into breast clinic.

Dedicated interventional radiology (IR) appointments were available in 27 (31.4%) hospitals, but GPs and ED staff were unable to book directly. In those units, where the provision of dedicated IR slots existed, surgical teams were expected to review the patient first (n=22, 25.6%). 13 (15.1%) hospitals allowed general surgeons to book patients directly into interventional radiology for urgent breast ultrasound scan (US) and US-guided intervention. 70 (81.4%) hospitals arranged breast US for all mastitis and breast abscess patients.

Criteria for hospital admission included: large abscess (36.1%), failure to respond to antibiotics (53.5%), sepsis (96.5%), immunocompromise (52.3%) or haemodynamic instability. (86.1%).

It was anticipated that most patients with breast abscesses were treated with US-guided needle aspiration (83 hospitals, 96.5%). Reported indications for surgical incision and drainage (I&D) were: skin changes or necrosis (93.0%), pointing (30.2%), size ≥5cm (17.4%) and multiloculated abscess (44.2%). 86.0% aimed to operate on the same day or the following day.

The most frequently recommended antibiotics were: flucloxacillin (86.1%), co-amoxiclav (61.6%), metronidazole (37.2%) and clindamycin (33.7%).

**Supplementary Appendixes**

## Data Collection for Phase 1

1. Local Mastitis / Breast abscess protocol present: Yes ☐ No ☐
2. Average number of Mastitis / Breast abscess cases seen at the Trust a month (excluding post-op surgical site infection following breast operation): _____
3. Patient treatment pathway:
   1. Predominant source of referral: A&E ☐ GP ☐ UCC ☐ Self-referral ☐ Maternity ☐
   2. Predominant location of treatment of breast abscess:
       A&E ☐ Inpatient ☐ Outpatient/Breast clinic ☐ Transferred to another centre ☐
   3. Predominant treatment team: General Surgical oncall team ☐ Breast team ☐
   4. Average waiting time for clinical review: Same day ☐ Next day ☐ Within 1 week ☐
   5. Breastfeeding support available: Post-natal ward ☐ Lactational specialist ☐
      Dual admission of mother and baby ☐ Patient information leaflet ☐
      Nil ☐ Don’t know ☐
4. Review by Breast surgeon / clinician/ associate specialist :
   All patients ☐ Selected patients ☐ Not routinely referred ☐
   1. Type of review: Inpatient ☐ Outpatient ☐ N/A ☐
   2. Waiting time to review: Same day ☐ Next day ☐ Within 1 week ☐
   3. Provision of ‘out of hours’ breast surgery cover: General Surgeons ☐ Breast Surgeons ☐
5. Dedicated breast clinic slots: Daily ☐ Weekly ☐ Nil ☐
   1. Direct GP access: Yes ☐ No ☐
   2. Direct A&E access: Yes ☐ No ☐
   3. Direct on-call team access: Yes ☐ No ☐
6. Dedicated interventional radiology clinic for breast infection: Yes ☐ No ☐
   1. Direct GP access: Yes ☐ No ☐
   2. Direct A&E access: Yes ☐ No ☐
   3. Direct on-call team access: Yes ☐ No ☐
   4. Patient must be seen by the breast team prior: Yes ☐ No ☐
7. Criteria for admission: All patients ☐ Large abscess ☐ No response to antibiotics ☐ Sepsis / Significant Infection ☐ Immunocompromised ☐ Haemodynamically unstable ☐ Other ________ ☐
8. Diagnostic Breast Ultrasound Scan:
   1. Performed in: All patients ☐ Selected patients ☐ Not routinely performed ☐
   2. If selected patients, what is the criteria for requesting USS: ______________
9. Primary mode of treatment for breast abscess (select one):
   US-guided aspiration ☐ Surgical Incision & Drainage ☐
10. Indication for surgical incision and drainage (select all applicable):
    Skin changes / necrosis ☐ Pointing ☐ Size ≥5cm ☐ Multiloculated abscess ☐ Duration of symptoms ≥5 days ☐ Other __________ ☐
11. Average waiting time for intervention: Same day ☐ Next day ☐ Within 1 week ☐
12. Antibiotics recommended on hospital guidelines (select all applicable):
    Erythromycin ☐ Flucloxacillin ☐ Dicloxacillin ☐ Amoxicillin ☐ Cephalexin ☐ Clindamycin ☐ Co-amoxiclav ☐ Vancomycin ☐ Metronidazole ☐

## Data Collection for Phase 2

**Patient Demographics**

1. Age (years): <20 ☐ 21-30 ☐ 31-40 ☐ 41-50 ☐ 51-60 ☐ 61-70 ☐
   71-80 ☐ >80 ☐
2. BMI: < 18.5 ☐ 18.5-24.9 ☐ 25-29.9 ☐ 30-39.9 ☐ >40 ☐
3. Postpartum: Yes ☐ No ☐
   1. Number of weeks since delivery: ______ weeks

**Patient treatment pathway**

1. Day of presentation: Weekday ☐ Friday ☐ Weekend ☐
2. Time of presentation: Day ☐ Evening ☐ Night ☐
3. First seen by: Breast team ☐ General surgical on call/take team ☐ A&E ☐
4. Source of referral to surgical / breast team: A&E ☐ GP ☐ Maternity services ☐
   Direct Self-referral ☐
   1. Did the patient see her GP prior to being seen in A&E: Yes ☐ No ☐
5. Antibiotics prior to being seen in hospital: Yes ☐ No ☐
   - 1. How many courses: 1 ☐ 2 ☐ ≥3 ☐
6. Breast surgeon/clinician/associate specialist review: Yes ☐ No ☐
   1. Inpatient ☐ Outpatient ☐
   2. Breast clinic follow-up: Yes ☐ No ☐

**Diagnosis**

1. Number of hours/days from onset of symptoms prior to seeking help: ____ hours/days
2. Diagnosis: Lactational Mastitis ☐ Lactational mastitis with Breast Abscess ☐
   Peri-ductal mastitis ☐ Peri-ductal mastitis with Breast Abscess ☐
   Peripheral non-lactational mastitis ☐
   Peripheral non-lactational mastitis with Breast Abscess ☐ Granulomatous mastitis ☐

**Risk Factors**

1. Breastfeeding: Yes ☐ No ☐
   1. Using breast pump regularly: Yes ☐ No ☐
2. Previous Breast infection (abscess / mastitis): Yes ☐ No ☐
3. Risk factors other than lactation: Smoking ☐ Diabetes ☐ Breast Trauma ☐
   Steroid Use ☐ IV drug use ☐ Recent Breast Intervention ☐ Co-morbidities: _______ ☐

**Treatment**

1. Advice to continue breastfeeding from the affected breast given: Yes ☐ No ☐ N/A ☐
2. Antibiotics prescribed at the hospital (list all): Yes ☐ No ☐
   1. Name: Erythromycin ☐ Flucloxacillin ☐ Dicloxacillin ☐ Amoxicillin ☐
      Cephalexin ☐ Clindamycin ☐ Co-amoxiclav ☐ Vancomycin ☐ Metronidazole ☐
   2. Route: Oral ☐ IV ☐
   3. Course duration (days): ≤7 ☐ 7-10 ☐ 10-14 ☐ >14 ☐
   4. Reason for choice of antibiotic: Hospital Protocol ☐ Patient Allergies ☐
      Previous treatment ☐ Other_________ ☐
3. Location of treatment: Inpatient ☐ Outpatient ☐
   1. Length of hospital stay: _______ days
   2. Reason for admission: severe infection/sepsis ☐ haemodynamically unstable ☐
      immunocompromised ☐ rapidly-progressing infection ☐ IV Antibiotics ☐
      Other ________ ☐
4. Decision made to admit by (select most senior decision-maker involved):
   Nurse practitioner ☐ HO/FY1 ☐ SHO/FY2/CT ☐ Registrar/>ST3 ☐ Consultant ☐
5. Diagnostic breast ultrasound scan: Yes ☐ No ☐
   1. Waiting time to diagnostic breast ultrasound scan : _______ days
6. Needle aspiration: Yes ☐ No ☐ N/A ☐
   1. Under ultrasound guidance: Yes ☐ No ☐
   2. Waiting time to 1^st^ needle aspiration: _______ days
   3. Number of aspirations performed in total: _______
7. Surgical Incision and drainage: Yes ☐ No ☐ N/A ☐
   1. Indication for surgical incision and drainage: Skin changes / necrosis ☐ Pointing ☐
      Size ≥5cm ☐ Multiloculated abscess ☐ Duration of symptoms ≥5 days ☐
      Other ________ ☐
   2. Waiting time to I&D: _______ days
   3. Returned to theatre for repeat I&D: Yes ☐ No ☐
8. Aspirate sent for culture and sensitivity: Yes ☐ No ☐ N/A ☐
   1. Pathogen isolated: Yes ☐ No ☐
   2. Pathogen: _______

**MAMMA Research Collaborative**

Ayesha Abbasi, Southend University Hospital

Rashad Abdelrahman, Lincoln County Hospital

Ahmed Ahmed, Royal Derby Hospital

Goran Ahmed, Frimley Park Hospital

Louise Alder, University Hospital Southampton

Badr Al-Khazaali, Princess Royal Hospital

Ghadah Alyahya, Leighton hospital

Verda Amin, Warwick Hospital

Aonghus Ansari, Leicester Glenfield Hospital

Tahera Arif, Wirral University Teaching Hospital

Deeksha Arora, Royal Derby Hospital

Laura Arthur, Royal Alexandra Hospital

Ed Babu, Hillingdon Hospital

Jenny Banks, Torbay and South Devon NHS Foundation Trust

Chwanrow Baban, Wexham Park Hospital

Zoe Barber, Princess of Wales Hospital

Alison Bate, Torbay and South Devon NHS Foundation Trust

Samuel Baxter, Queen Elizabeth Hospital, Gateshead

Sumbal Bhatti, Norfolk and Norwich University Hospital

Raouef Ahmed Bichoo, Hull University Teaching Hospitals

Kimberley Bossi, Frimley Park Hospital

Alexander Boucher, King's Mill Hospital

Arjuna Brodie, Leicester Glenfield Hospital

Gwen Bromley, Queen Elizabeth Hospital, Gateshead

Eilidh Bruce, Aberdeen Royal Infirmary

Katie Campbell, Wythenshawe hospital

Alice Chambers, North Bristol NHS Trust, Southmead Hospital

Despoina Chatzopoulou, Frimley Park Hospital

Qian Chen, Frimley Park Hospital

Zoe Chia, Nottingham University Hospital NHS Trust

Sharat Chopra, Cardiff and Vale University Health Board

Nicola Cook, Great Western Hospital, Swindon

Carolyn Cullinane, St Vincent’s University Hospital, Ireland2

Marta D’Auria, Lincoln County Hospital

Katherine De Rome, West Middlesex Hospital

Michael Devine, University Hospital Limerick

Candice Downey, Airedale General Hospital

Ghassan Elamin, King’s Mill Hospital

Sabreen Elbakri, Ninewells hospital

Kenneth Elder, Western General, Edinburgh

Ibrahim Elzayat, Poole Hospital

Buket Ertansel, St George’s Hospital

Hana Esack, Lincoln County Hospital

Katherine Fairhurst, Royal United Hospitals, Bath

Natalie Fairhurst, University College London Hospital

Kyrllos Farag, West Suffolk Hospital

Michael Flanagan, University Hospital Waterford

Joey Fong, St Helens and Knowsley NHS Trust

Rachel Foster, Countess of Chester Hospital NHS Trust

Anna Fullard, University Hospital Galway, Ireland

Katie Gilmore, Royal United Hospital Bath

Tasha Gandamihardja, Broomfield Hospital

Jayan George, Sheffield Teaching Hospitals NHS Foundation Trust, Royal Hallamshire Hospital

Sarah Gibbins, St James's University Hospital

Lucy Gossling, Royal Derby Hospital

Tabitha Grainger, Charing Cross Hospital

Tomasz Graja, Dorset County Hospital Foundation Trust

Lauren Hackney, Belfast City Hospital

Hytham K. S. Hamid, William Harvey Hospital

Ishita Handa, University Hospital Southampton

Grant Harris, Northumbria NHS Foundation Trust

Muhammad Hashmi, Dorset County Hospital Foundation Trust

Natalie Hirst, Sheffield Teaching Hospitals NHS Foundation Trust

Nicholas Holford, Charing Cross Hospital

Ciaran Hollywood, Chesterfield Royal Hospital

Thomas Hubbard, Royal Devon and Exeter Hospital

Gemma Hughes, Northampton General Hospitals

Anna Isaac, Belfast City Hospital

Javeria Iqbal, Leicester Glenfield Hospital

Urvashi Jain, Guys and St Thomas' NHS Foundation Trust

Sam Jeffreys, Royal Glamorgan Hospital, Llantrisant

Michael Jones, Cheltenham General Hospital

Charlotte Kallaway, Frimley Park Hospital

Trisha Kanani, Nottingham University Hospital NHS Trust

Georgios Karagiannidis, Ipswich Hospital

Rahi Karmarkar, Princess of Wales Hospital

Emma Kellett, Hull University Teaching Hospitals

Michael Kelly, West Middlesex University Hospital

Eiman Khalifa, Castle Hill Hospital

Eleftheria Kleidi, Cambridge University Hospitals

Grace Knudsen, Frimley Park Hospital

Akanksha Kiran, Dorset County Hospital Foundation Trust

Katharine Kirkpatrick, Bedfordshire Hospitals

Tamara Kiernan, St Helens and Knowsley NHS Trust

Amanda Koh, Nottingham University Hospital NHS Trust

Anneliese Lawn, Ashford and St Peter's Hospital

Alice Lee, West Middlesex University Hospital

Rachel Lee, Royal Derby Hospital

Harry Yeuk Hei Lei, Charing Cross Hospital

Rebecca Llewellyn-Bennett, Cheltenham General Hospital

Jennifer Long, Royal Glamorgan Hospital, Llantrisant

Joseph Maalo, West Herts Hospitals NHS Trust

Kiran Majid, Royal Derby Hospital

Francesca Malcolm, Chesterfield Royal Hospital

Loaie Maraqa, Royal Hallamshire Hospital

Gabriella Marchitelli, Royal Victoria Infirmary

Hannah Markey, University Hospital Galway, Ireland

Josh Marston, Hull University Teaching Hospitals

Julia Massey, Chesterfield Royal Hospital

Colin McIlmunn, Belfast City Hospital

Radhika Merh, Maidstone and Tunbridge Wells

Evangelos Mallidis, Ipswich Hospital

Diya Mirghani, Cumberland Infirmary Carlisle

Bahar Mirshekar-Syahkal, West Suffolk Hospital

Jenna Morgan, Doncaster Royal Infirmary

Samantha Muktar, Addenbrooke’s Hospital

Francesk Mulita, General University Hospital of Patras

Brenda Muntean, Queens Hospital Burton

Titus Murphy, Guy's and St Thomas' NHS Trust

Erum Najeeb, Derriford Hospital, Plymouth

Vijay Narbad, King's Mill Hospital

Hedwige Nathaniel, Northwick Park Hospital

George Neelankavil Davis, Dorset Couny Hospital

Eleni Ntakomyti, University College London Hospital

Nur Nurmahomed, Chelsea and Westminster Hospital

Olaniyi Olayinka, Barnsley Hospital NHS Foundation Trust

Elaf Osman, University Hospital Waterford, Ireland

Olamide Oyende, Nottingham University Hospital NHS Trust

Benjamin Patel, North Bristol NHS Trust, Southmead Hospital

Thalia Picton-Scott, St George's Hospital

Simon Pilgrim, Leicester Glenfield Hospital

Heather Pringle, Royal Devon and Exeter Hospital

Shahnaz Qureshi, Northwick Park Hospital

Lilia Ragad, Princess Royal University Hospital

Jaideep Rait, Maidstone & Tunbridge Wells NHS Trust

Ritika Rampal, Hull University Teaching Hospitals

Sabina Rashid, Northwick Park Hospital

Shiveta Razdan, Wexham Hospital, Frimley Health

Monica Reeves, St Helens and Knowsley NHS Trust

Monika Rezacova, Poole General Hospiral

Azel Regan, Nevill Hall Hospital

Alexander Ribbits, Conquest Hospital

Bahaty Riogi, St Helens & Knowsley Teaching Hospitals

Henry D Robb, Charing Cross Hospital

Clare Roger, Doncaster & Bassetlaw Teaching Hospitals

Catherine Rossborough, Craigavon Area Hospital

Fiona Rutherford, Royal Alexandra Hospital

Sunita Saha, Colchester General Hospital

Giovanni Santoro, Countess of Chester Hospital NHS Trust

Norah Scally, Craigavon Area Hospital

Tom Seddon, Kettering Hospital

Ashvina Segaran, Thames Valley

Syed Noor Hussain Shah, Bon Secours Hospital, Ireland

Ahmed Shalaby, Buckingham Healthcare Trust

Anita Sharma, Torbay and South Devon NHS Foundation Trust

Rishabha Sharma, Royal united Hospital, Bath

Joshua Silva, Charing Cross Hospital

Chiara Sirianni, Betsi Cadwaleadr UHB West

Noyko Stanilov, University College London Hospital

Joanna Stringer, Northampton General Hospital

Sreekumar Sundara Rajan, Nottingham University Hospitals NHS Trust

Arthika Surendran, University Hospital Coventry & Warwickshire

Khalida Suri, University College London Hospital

Alexandra Tenovici, Wexham Hospital, Frimley Health

Róisín Tully, Cork University Hospital

Marina Verebcean, Diana Princess of Wales Hospital Grimsby

Rashmi Verma, Royal Bolton Hospital

Livia Walsh, Ashford and St Peter’s Hospitals NHS Foundation Trust

Anjelli Wignakumar, Colchester General Hospital

Myat Win, West Hertfordshire Hospitals NHS Trust

Jih Dar Yau, Hull University Teaching Hospitals

**Acknowledgments**

Mohammed Absar, North Manchester General Hospital

Deepika Akolekar, Maidstone and Tunbridge Wells

Dibyesh Banerjee, St George's Hospital

Henry Cain, Royal Victoria Infirmary

Karina Cox, Maidstone & Tunbridge Wells NHS Trust

Mark Corrigan, Cork University Hospital

Thekkinkattil Dinesh, Lincoln County Hospital

Abigail Evans, Poole Hospital

Amit Goyal, Royal Derby Hospital

Tomasz Graja, Dorset County Hospital Foundation Trust

Kartikae Grover, Hull University Teaching Hospital

Richard Hunt, Cheltenham General Hospital

Sandeep Joglekar, James Paget University Hospital

Isabella Karat, Frimley Park Hospital

Tamara Kiernan, St Helens and Knowsley NHS Trust

Beak Kim, St James's University Hospital, Leeds

Cliona Kirwan, Wythenshawe hospital

Aoife Lowery, University Hospital Galway

Evangelos Mallidis, Ipswich Hospital

Jamie McIntosh, Royal united Hospital, Bath

Robert Milligan, Queen Elizabeth Hospital, Gateshead

Gerry O’Donoghue, University Hospital Waterford

Gary Osborn, Royal Glamorgan Hospital, Llantrisant

Vasileios Pitsinis, NHS Tayside (Ninewells)

Jacqueline Rees-Lee, Torbay and South Devon NHS Foundation Trust

Walid Sasi, Leicester Glenfield Hospital

Elizabeth Shah, Conquest Hospital

Jennifer Smith, Diana Princess of Wales Hospital Grimsby

Ioannou Stella, Cumberland Infirmary

Sarah Tang, St George’s Hospital

Alexandra Tenovici, Wexham Hospital, Frimley Health

Lisa Whisker, Nottingham University Hospital NHS Trust

Samantha Williams, Great Western Hospital

Brendon Wooler, Hull University Teaching Hospital

Magdi Youssef, Northumbria NHS Foundation Trust

Mina Youssef, Norfolk and Norwich University Hospital

**Supplementary Figures and Tables**

## Table S1. Timing of presentation.

|  | **n (%)** |
| --- | --- |
| **Day of presentation** | |
| **Weekday (Monday-Thursday)** | 1 021 (77.8) |
| **Friday** | 132 (10.1) |
| **Weekend** | 159 (12.1) |
| **Time of presentation** | |
| **Day (8.00-17.00)** | 1 064 (81.1) |
| **Evening (17.00-20.00)** | 126 (9.6) |
| **Night (20.00-8.00)** | 120 (9.1) |

Data was missing for 2 patients for time of presentation.

## Figure S1. Median age of patients at the time of diagnosis.

##


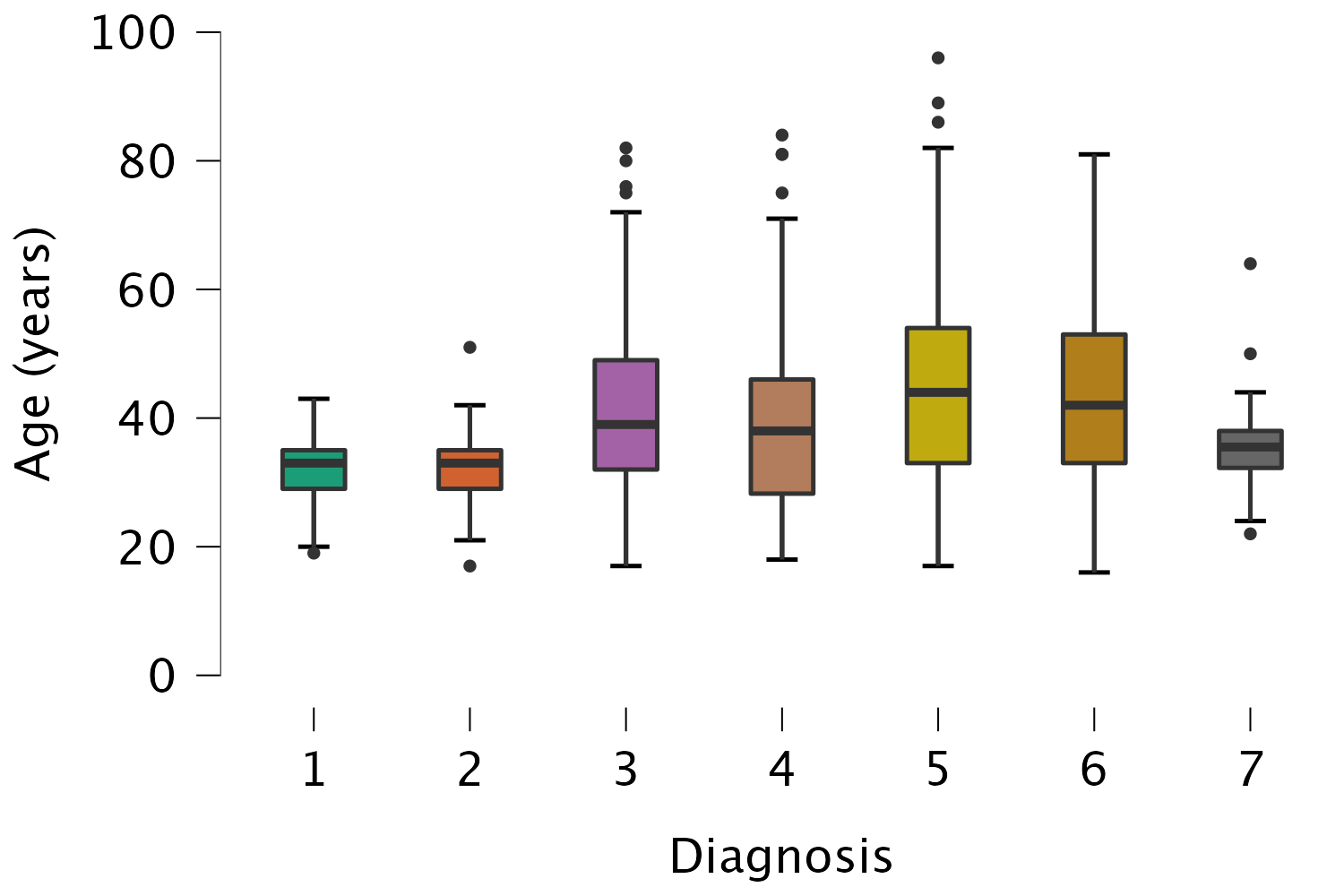

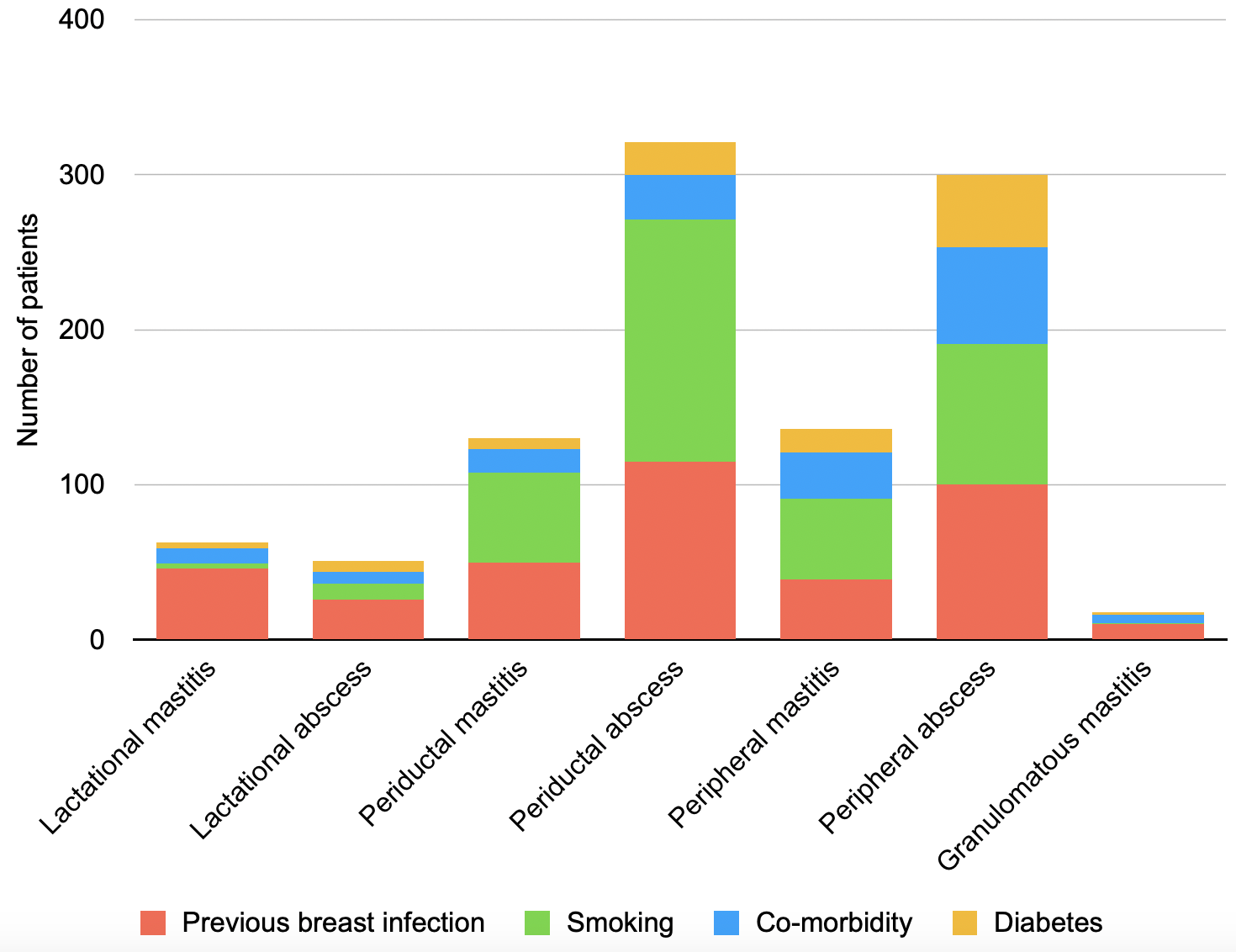


Significant difference was observed between the median age of patients presenting with lactational and non-lactational disease (lactational mastitis / abscesses cf. periductal / peripheral mastitis / abscesses) with the exception of granulomatous mastitis (Kruskal-Wallis test, H=185.0, p<0.001).

## Figure S2. Body mass index of patients.


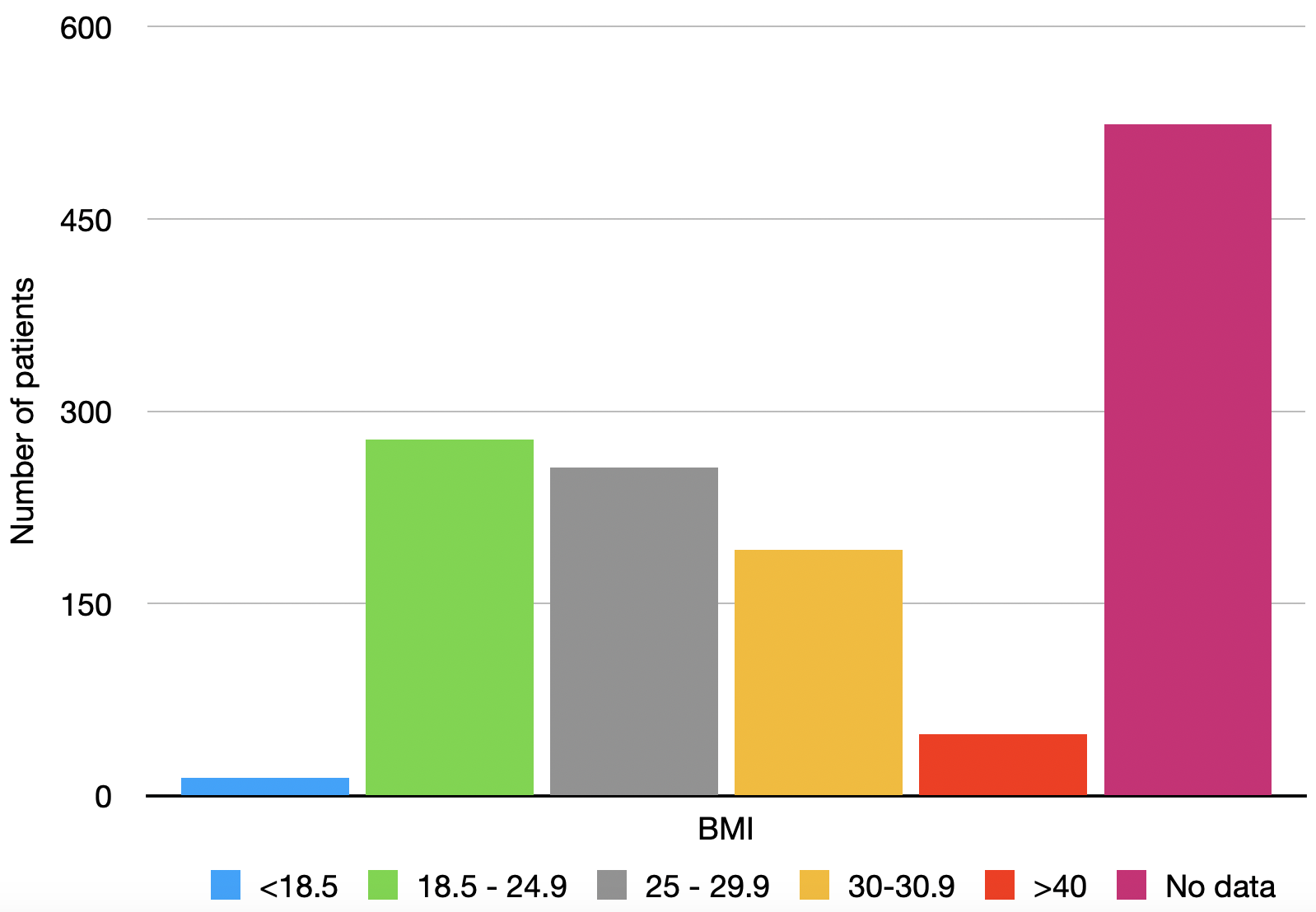


Body mass index (BMI) was calculated as weight (kg) over height squared (m^2^). Data was missing for 524 patients included in this audit. At least a third of patients were overweight (BMI>25).

**Figure S3. Relationship between the risk factors and the type of mastitis or breast abscess.**


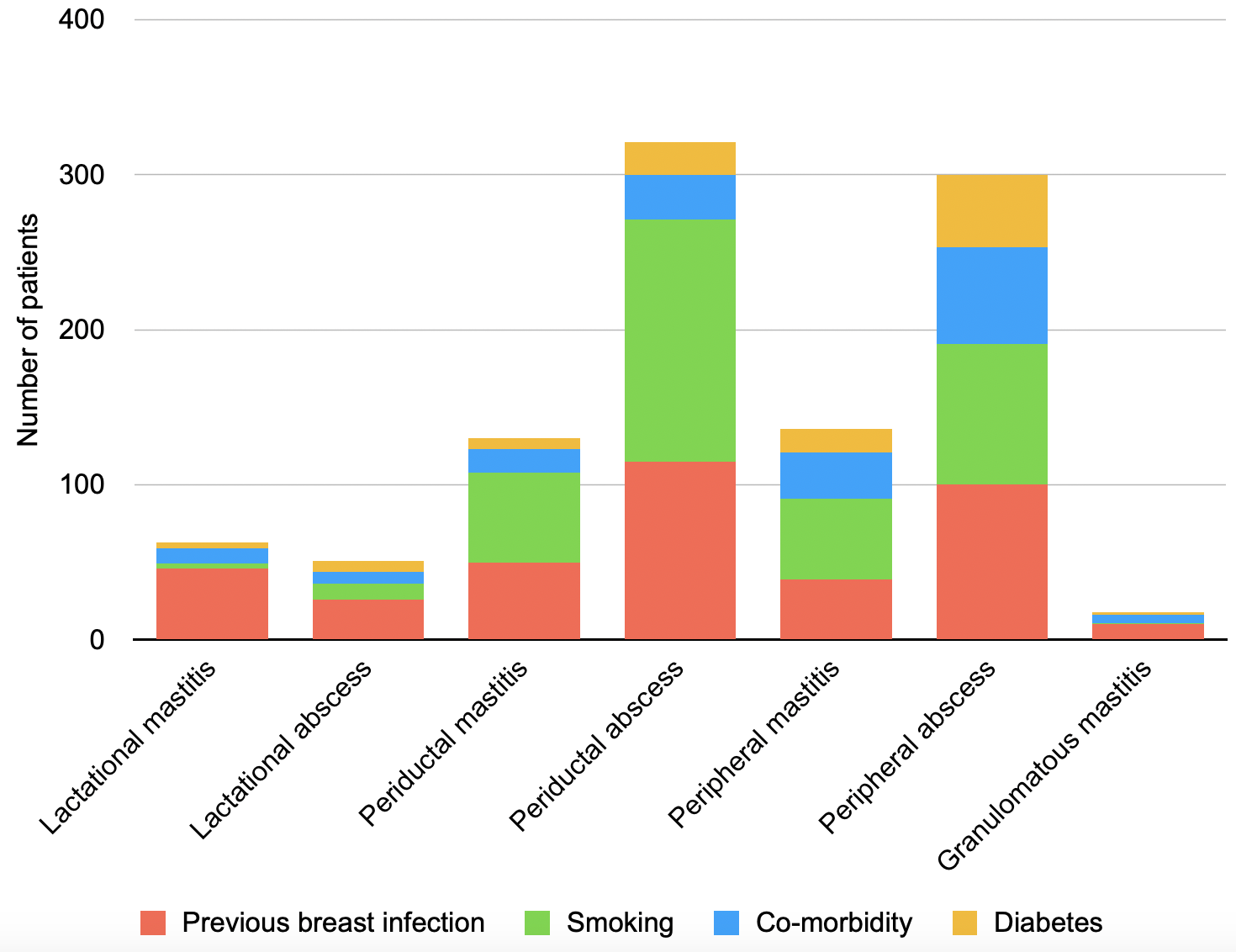


Bars represent the number of patients with a risk factor.

## Figure S4. Frequency of the risk factors for development of mastitis and breast abscesses.


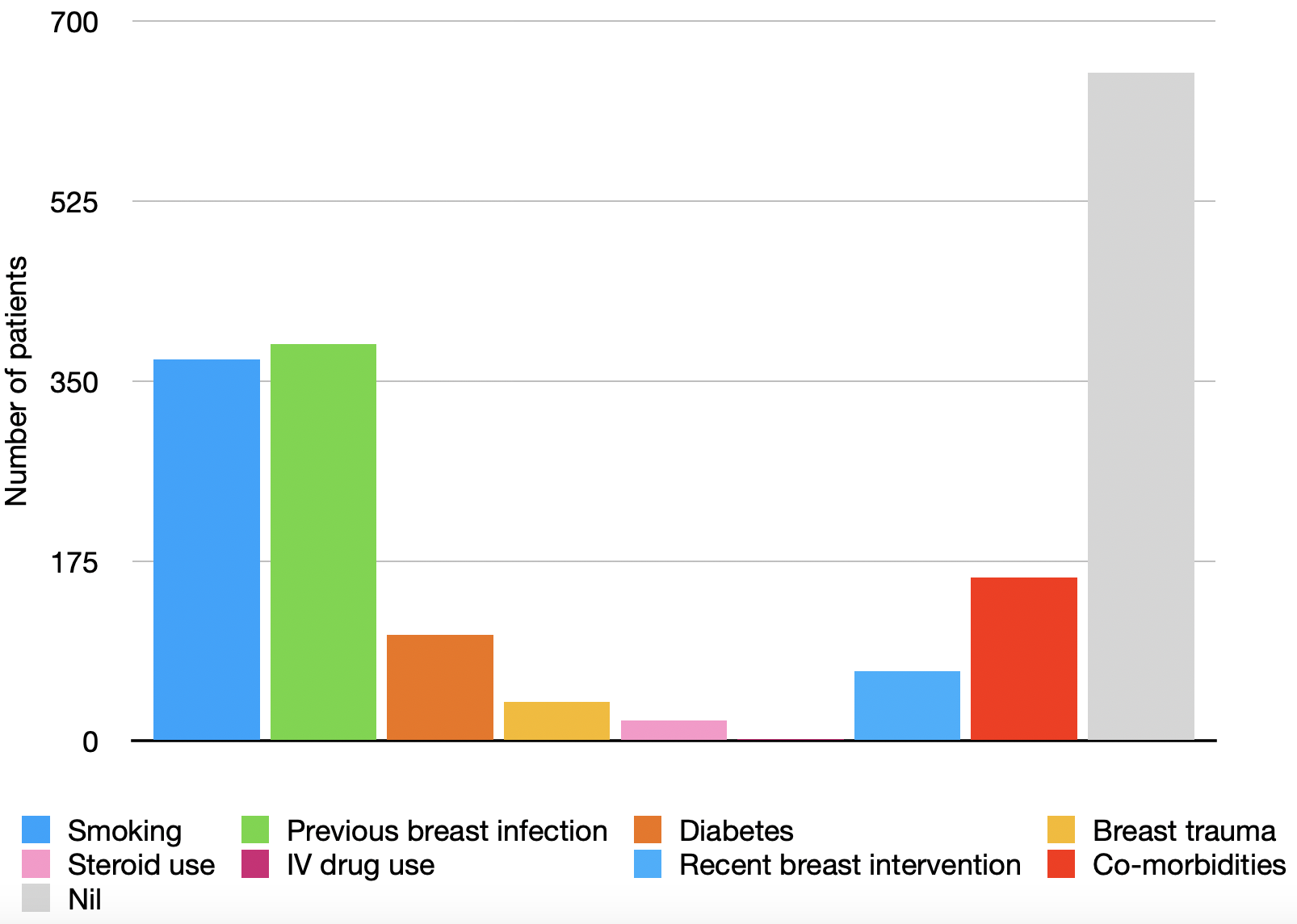


The most common observed risk factor was smoking. About half of all patients did not have any risk factors.

## Figure S5. Timing of presentation.

##
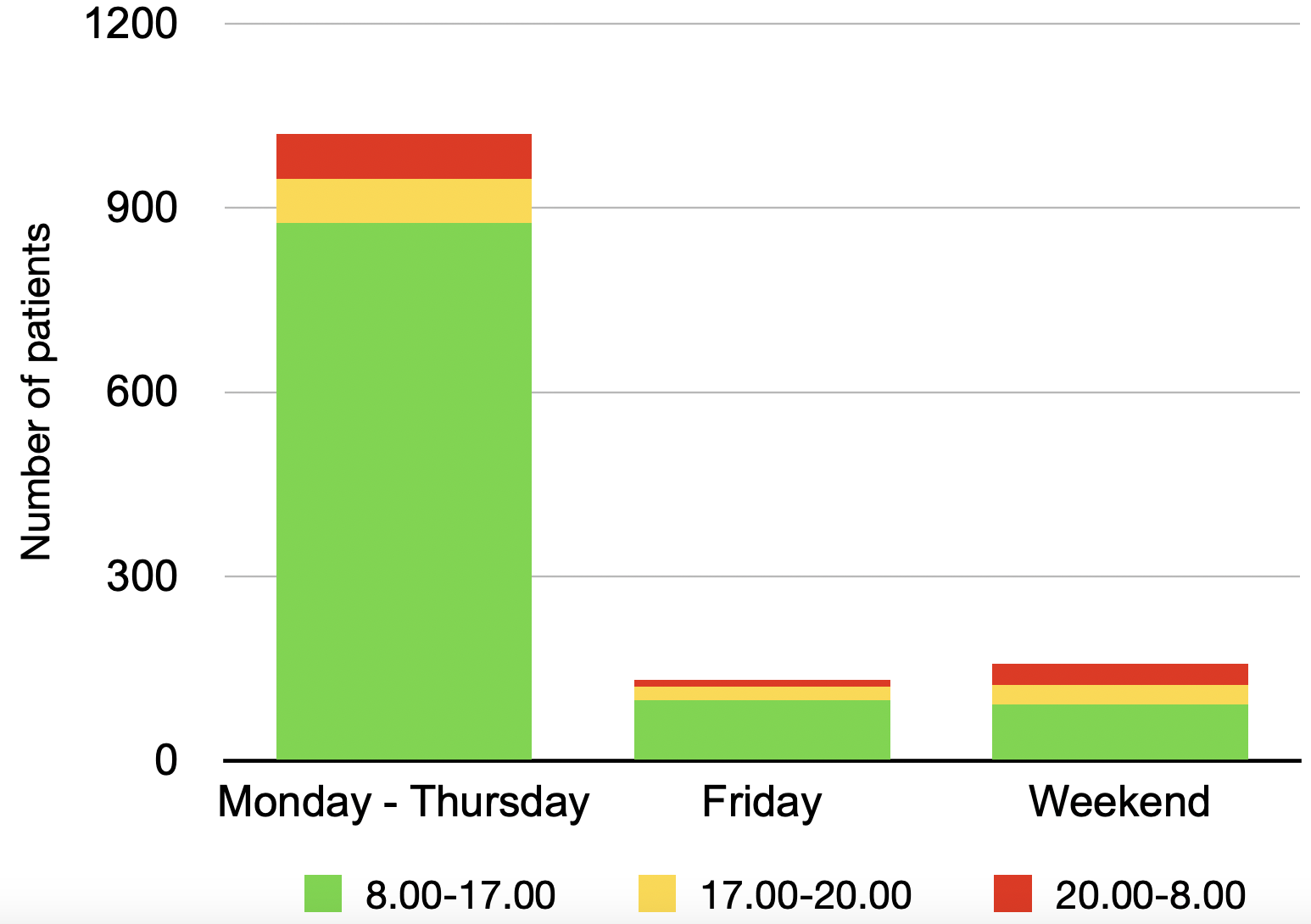


Most patients presented between 8 o’clock in the morning and 5 o’clock in the afternoon on Monday to Thursday.

## Figure S6. Relationship between the a) day of presentation and b) time of presentation and the source of referral.


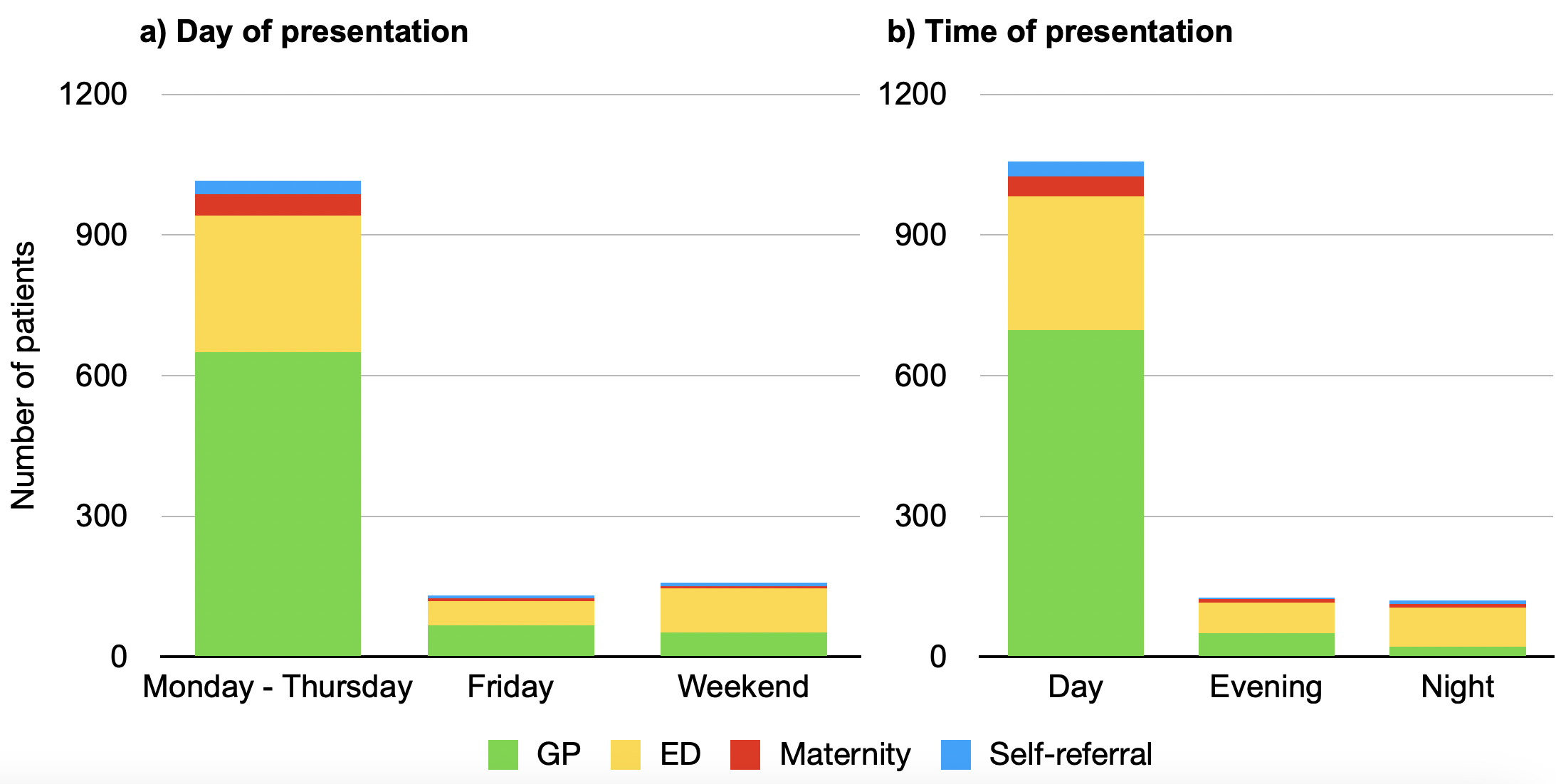


Less than half of all patients were seen by the breast team directly, even during normal working hours between Monday and Thursday. Although the proportion of patients presenting on Friday and at weekends was much smaller, most of these patients were seen by the on-call general surgery or emergency department (ED) team first.

## Figure S7. Relationship between the a) day of presentation and b) time of presentation and the seniority of decision maker.


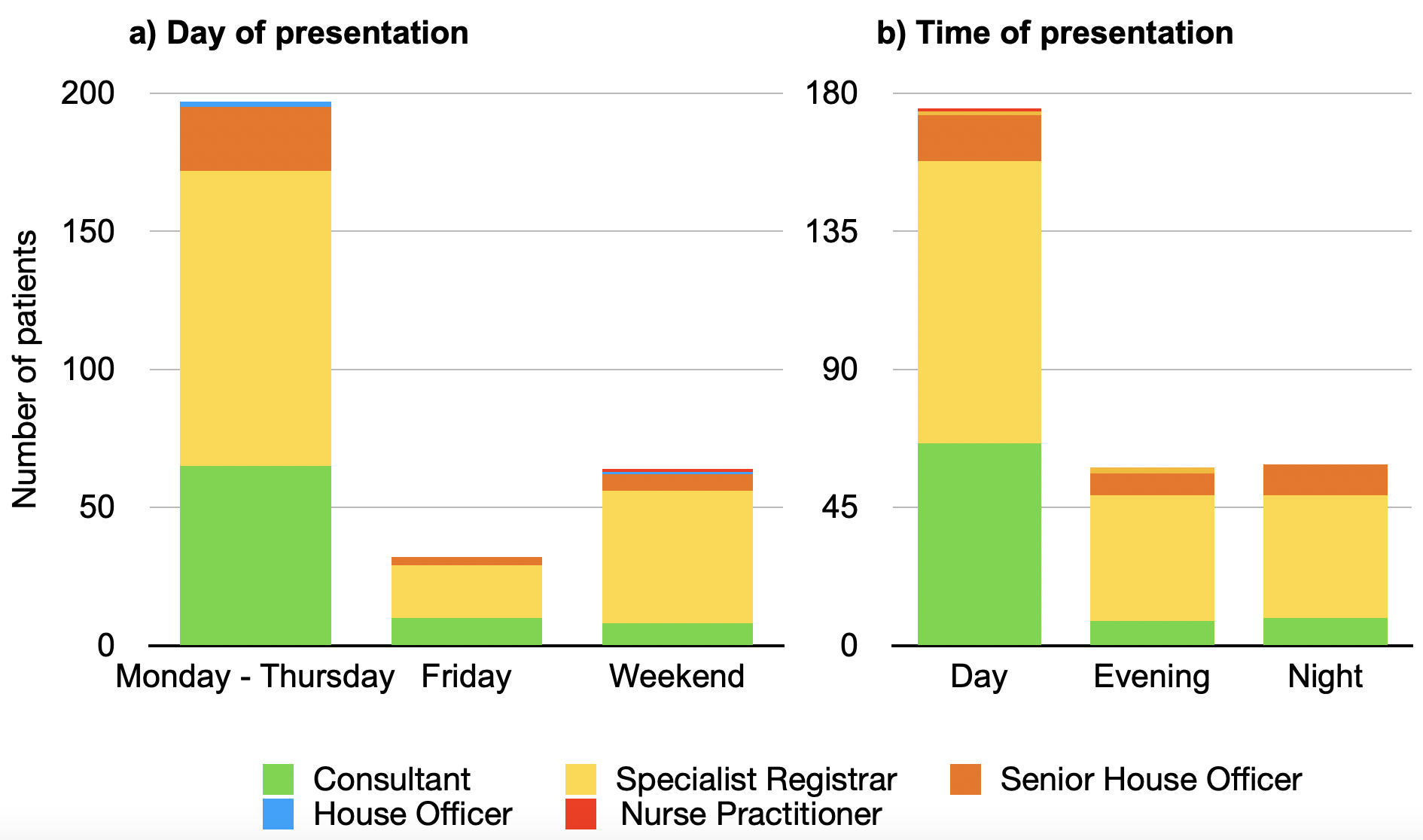


295 patients were admitted for inpatient treatment; data was available for 292 patients. Decision to admit was predominantly made by the specialist registrars, regardless of the day or time of presentation.

## Figure S8. Choice of antibiotics in the secondary care.


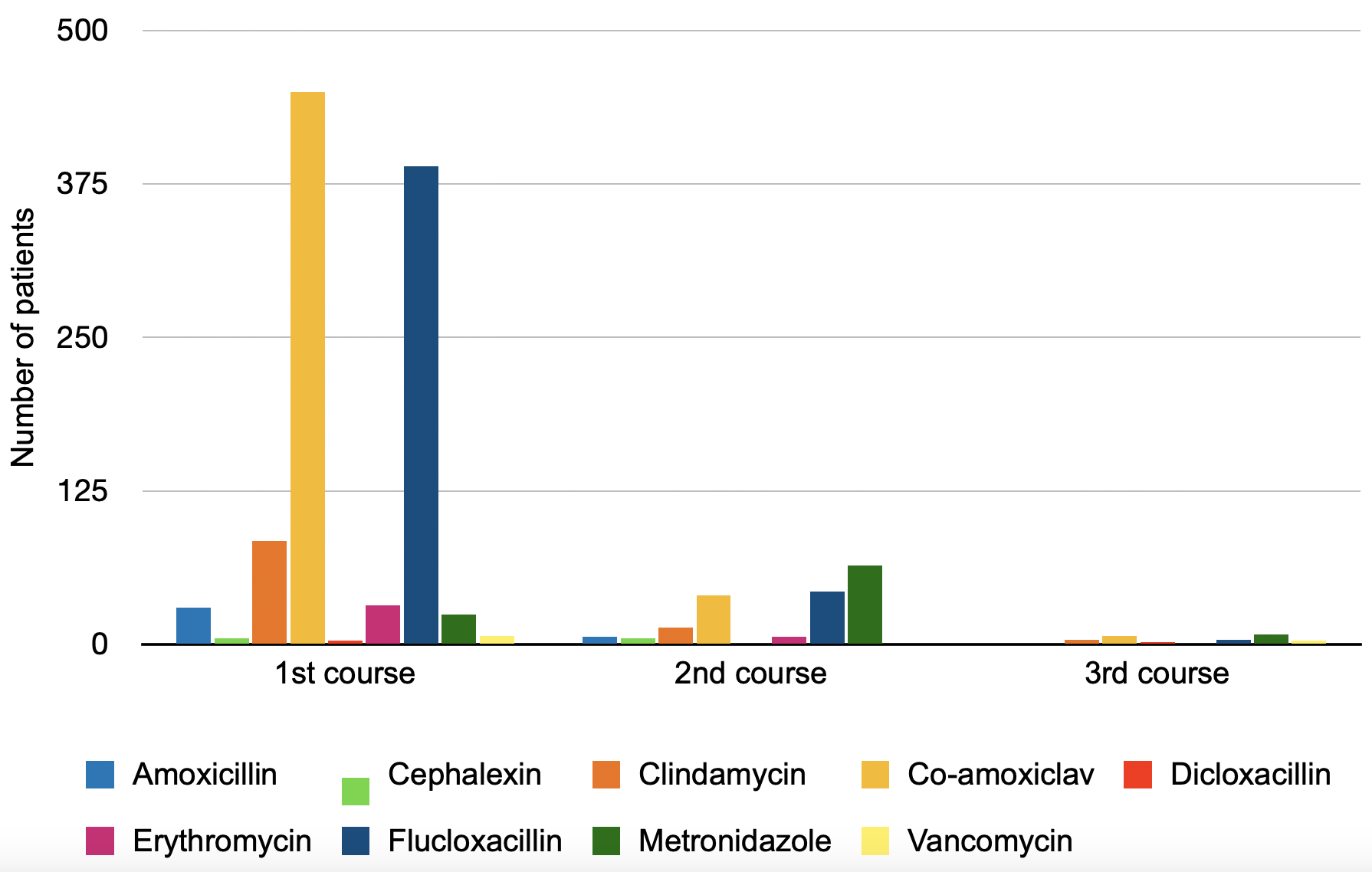


1 055 patients were prescribed antibiotics in the secondary care. Co-amoxiclav was the most frequently prescribed antibiotic as the first line treatment. Metronidazole was the most commonly selected antibiotic as the second line treatment. Only a minority of patients (n=33) required three or more courses.
